# DBPP-Predictor: a novel strategy for prediction of chemical drug-likeness based on property profiles

**DOI:** 10.1186/s13321-024-00800-9

**Published:** 2024-01-05

**Authors:** Yaxin Gu, Yimeng Wang, Keyun Zhu, Weihua Li, Guixia Liu, Yun Tang

**Affiliations:** Shanghai Frontiers Science Center of Optogenetic Techniques for Cell Metabolism, Shanghai Key Laboratory of New Drug Design, School of Pharmacy, East China University of Science and Technology, Shanghai, 200237 China

## Abstract

Evaluation of chemical drug-likeness is essential for the discovery of high-quality drug candidates while avoiding unwarranted biological and clinical trial costs. A high-quality drug candidate should have promising drug-like properties, including pharmacological activity, suitable physicochemical and ADMET properties. Hence, in silico prediction of chemical drug-likeness has been proposed while being a challenging task. Although several prediction models have been developed to assess chemical drug-likeness, they have such drawbacks as sample dependence and poor interpretability. In this study, we developed a novel strategy, named DBPP-Predictor, to predict chemical drug-likeness based on property profile representation by integrating physicochemical and ADMET properties. The results demonstrated that DBPP-Predictor exhibited considerable generalization capability with AUC (area under the curve) values from 0.817 to 0.913 on external validation sets. In terms of application feasibility analysis, the results indicated that DBPP-Predictor not only demonstrated consistent and reasonable scoring performance on different data sets, but also was able to guide structural optimization. Moreover, it offered a new drug-likeness assessment perspective, without significant linear correlation with existing methods. We also developed a free standalone software for users to make drug-likeness prediction and property profile visualization for their compounds of interest. In summary, our DBPP-Predictor provided a valuable tool for the prediction of chemical drug-likeness, helping to identify appropriate drug candidates for further development.

**Supplementary Information:**

The online version contains supplementary material available at 10.1186/s13321-024-00800-9.

## Introduction

**Fig. 1:**
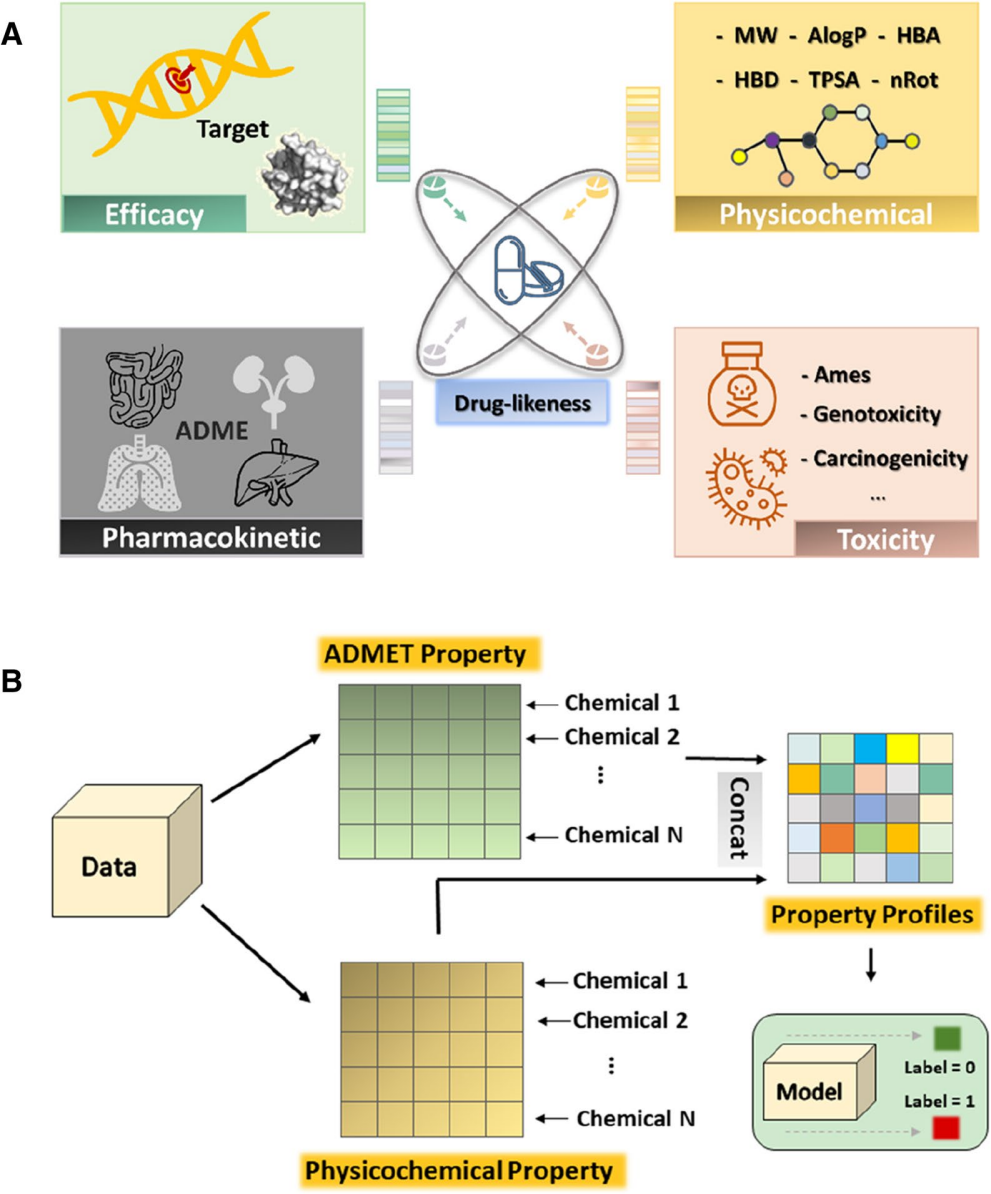
**A** The critical factors affecting drug-likeness. Chemical drug-likeness is the desirable characteristics to become a drug, including appropriate physicochemical, biochemical and pharmacokinetic properties, as well as safety. **B** Diagram of drug-likeness prediction based on property profiles (DBPP-Predictor). The property profile of a molecule consists of its physicochemical property profile and ADMET property profile

Chemical drug-likeness means the possibility of a compound to become a real drug. An ideal drug-likeness of a compound should be a balance among safety, efficacy, and pharmacokinetic properties (Fig. 1A) [1–3]. Despite significant advances in drug discovery and development technology in recent years, poor pharmacokinetic properties or safety are still the major causes of drug failures [4–6]. Therefore, it is a good idea to evaluate the drug-likeness of a compound at the very early stage of drug discovery, in order to reduce the attrition rate.

However, it is usually a challenging task to evaluate the drug-likeness of a drug candidate [7]. Traditionally, drug-like molecules are determined by experiments, which are costly, time-consuming and laborious. Therefore, computational methods have been developed to identify drug-like molecules [8–10]. The earliest efforts could be back to the 1990s, when the rule-of-five (Ro5) [11] was presented by Lipinski et al. based on statistical analysis of 2245 drugs from the World Drug Index (WDI). Later, Muegge et al. proposed a method to define drug-like molecules in terms of functional groups [12]. Though these rules of thumb were questioned recently [13–15], they paved the way for the development of more comprehensive drug-like indicators. A representative work, the quantitative estimate of drug-likeness (QED) [16], was proposed by Bickerton et al. in 2012, which assessed drug-likeness of a compound as a quantitative score by fitting the distribution of eight properties. In 2019, we defined a scoring function namely ADMET-score [17] for drug-like assessment by integrating 18 properties of compounds. Nevertheless, these methods only relied on drugs rather than non-drugs, hence it was hard to differentiate drug-like molecules from non-drug-like ones [18, 19].

More recently, machine learning (ML) models were developed to discriminate drugs from non-drugs. The combination of extended connectivity fingerprints (ECFPs) and support vector machine (SVM) was reported to significantly improve the accuracy in prediction of drug-like molecules [20]. Considering that hand-crafted features could be limited by large-scale screening, deep learning (DL) methods were utilized. Sun et al. [21] introduced a graph convolutional attention network (D-GCAN) to aid in screening potential inhibitors of the SARS-CoV-2 3C-like protease. In a separate study, Cai et al. [22] employed an active ensemble learning strategy to investigate drug-likeness prediction at a more subdivisional level. Beker et al. [23] evaluated different drug-likeness models with uncertain quantification from Bayesian neural networks. These binary methods were reported to rely on modeling data and had poor generalization abilities in real-world samples. Recently, a recurrent neural network-based language model [24] was designed for drug-like scoring in an unsupervised scenario, which was independent of negative samples and provided a new perspective on drug-like scoring design. However, it is worth highlighting that to improve filter efficiency while to enhance model interpretability is also critical for the prediction of drug-likeness. More than just drug-likeness prediction, it is meaningful to provide optimization guidance for molecules with poor drug-likeness.

In this study, we developed a property profile-based prediction strategy, namely DBPP-Predictor, for efficient assessment of chemical drug-likeness. DBPP-Predictor incorporated ML framework with important physicochemical and ADMET (absorption, distribution, metabolism, excretion, and toxicity) properties closely related to drug-likeness. It extracted feasible molecular representations in a data-driven manner. Compared with classical molecular representations, the property profile-based strategy has enhanced robustness and generalizability. In addition, it demonstrated mild sample dependence. DBPP-Predictor displayed promising identification potential across different data sets and was expected to provide a plausible and valuable drug-likeness scoring tool for virtual screening. The development of user-friendly stand-alone software facilitated support for drug-likeness prediction and visualization of property profiles.

## Materials and methods

### Data collection and preparation

The known small molecular drugs were considered as positive data. Drugs approved by the U.S. Food and Drug Administration (FDA_drug) and the other approved drugs (Worlddrug) were collected, respectively. Beker et al. [23] evaluated ZINC [25] as the "most likely non-drug data set" and recommended it as an efficient negative sample set. In addition to ZINC, non-drug sets were prepared from diverse databases, including ChEMBL [26] and GDB17 [27], for assessing the generalization ability. The positive unlabeled learning (PU learning) approach proposed by Liu et al. [28] was used to explore the effect of data noise. Meanwhile, the down-sampling strategy was employed to avoid the data imbalance problem. To alleviate the effects of data dependence, random down-sampling was performed three times in parallel.

For further assessing the feasibility, the drug-likeness of the three data sets was explored: the withdrawn drug set (WITHDRAWN) [29], the investigation group of DrugBank database [30] (Investigation), and the natural product set from TCMSP database [31] (TCMSP). Data preparation process was as follows: (1) Salts were converted to the corresponding acids or bases. (2) Mixtures and inorganic substances were removed. (3) Standardized SMILES strings and duplicate molecules were removed.

### Molecular representation

#### Molecular descriptors

In this study, each molecule was assigned a vector containing 200 molecular descriptors derived from the DescriptaStorus package (https://github.com/bp-kelley/descriptastorus). Normalized and non-normalized forms of descriptor representations were set up. The effect of feature scaling on the descriptor representation was explored.

#### Molecular fingerprints

A total of five molecular fingerprints were used to represent the compounds in this study. They were generated by RDKit package (Version 2021.03.4), including MACCS fingerprint (MACCS, 166 bits), Morgan fingerprint (Morgan, 2048 bits), AtomPairs fingerprint (AtomPairs, 2048 bits), RDK fingerprint (RDKFingerprint, 2048 bits), TopoTorsion fingerprint (TopoTorsion, 2048 bits).

#### Molecular graphs

In graph representation, the input compound was considered as a molecular graph, with atoms being the nodes and chemical bonds being the edges. The smiles_to_bigraph module of Deep Graph Library [32] was used to generate molecular graphs from the SMILES strings. The node and edge features of the compounds were extracted by the RDKit package. The initial atomic and bond features were shown in the Additional file 1: Tables S1–S4.

#### Property profiles

ADMET and physicochemical properties play key roles in drug-likeness evaluation. In this study, property profile-based drug-likeness was introduced to characterize the compounds. The property profile of each compound was a 26-bit property description vector obtained from the drug-likeness related property endpoints. Figure 1B depicted the overall scheme of DBPP-Predictor for the prediction of chemical drug-likeness.

Properties closely related to the drug-likeness were hybridized to obtain a property profile representation. A weighting parameter γ was introduced to adjust the combination weights, taking values from 0 to 1. The formula is as follows:

1 $$\mathrm{Property \,Profile}={\text{Concat}}(\left(2-2\upgamma \right){\text{PC}}, 2\mathrm{\gamma ADMET})$$

where PC stands for physicochemical properties and ADMET means ADMET properties.

### Machine learning approaches

Three machine learning algorithms were adopted to construct prediction models, including logistic regression (LR), support vector machine (SVM) and LightGBM. LR is a generalized linear model with the features of simplicity, parallelizability and interpretability [33]. It is a classical algorithm for binary classification. SVM finds an optimal hyperplane to distinguish samples and constructs a discriminative classifier. It solves the linear indivisibility problem by introducing different kernel functions to achieve a high-dimensional mapping of the input vectors [34]. The regularization parameter C is one of the important parameters to be optimized by the SVM. Both LR and SVM were performed via the scikit-learn package. LightGBM is a faster, less memory-consuming and more accurate gradient enhancement framework [35]. The max_depth and num_leaves parameters are optimized. LightGBM models were supported by the LightGBM package (https://github.com/microsoft/LightGBM). The GridSearchCV tool in the scikit-learn package was used to find the proper parameters for each model.

### Graph neural network approaches

In addition to conventional machine learning algorithms, four graph neural network (GNN) architectures were employed for drug-likeness assessment, including graph convolutional network (GCN), graph attention network (GAT), graph sample and aggregate (GraphSAGE) [36], and AttentiveFP network. GCN was proposed in 2017 [37], using convolution for graph data feature extraction. Message passing and readout are two phases present in the forward propagation process. For graph classification tasks, the central atom aggregates the information of neighboring nodes through iterative updates of the state. In the readout phase, atomic representations are aggregated for property prediction. GAT is an extension of GCN that introduces an attention mechanism for updating node representations [38], while GraphSAGE updates the embedding of nodes by subgraphs. Attention mechanisms are incorporated at the atomic and molecular levels to aid in the learning of local and global features [39], respectively. It effectively captures the non-local features of the graph and the interactions of distant nodes.

All GNN models were built with the Deep Graph Library (DGL) [32] package (version 0.7.0) and PyTorch [40] framework (version 1.8.1). The model parameters were using the Adam [41] optimizer for gradient descent optimization. BCEWithLogitsLoss was set as the loss function for the binary classification tasks. Bayesian optimization [42] was used to obtain the proper hyperparameters for the GNN models, such as learning rate, weight decay, batch size, and so on. To avoid overfitting and save training resources, the early stopping strategy was used during the training process.

### Performance evaluation

Ten-fold cross-validation and external validation were utilized for model evaluation. We used the area under the receiver operating characteristic (ROC) curve to analysis, which was plotted by the true prediction rate (TPR) against the false positive rate (FPR). The area under the ROC curve (AUC) was calculated for each model to exhibit the performance of the classification models. In addition, to further evaluate the performance of the model, four statistical metrics were used, including accuracy, recall, specificity (SP), and precision, which were defined as follows:

2 $$Accuracy=\frac{{\text{TP}}+{\text{TN}}}{{\text{TP}}+{\text{TN}}+{\text{FP}}+{\text{FN}}}$$

3 $$Recall=\frac{{\text{TP}}}{{\text{TP}}+{\text{FN}}}$$

4 $$SP=\frac{{\text{TN}}}{{\text{TN}}+{\text{FP}}}$$

5 $$Precision=\frac{{\text{TP}}}{{\text{TP}}+{\text{FP}}}$$

where TP: true positive; TN: true negative; FP: false positive; FN: false negative.

### Development of standalone software

A standalone software called DBPP-Predictor was developed via Tkinter [43]. The optimal DBPP-Predictor model was encapsulated in the software. The software includes two major functional modules, drug-likeness assessment and property profile visualization. The drug-likeness assessment module supports single molecule and batch molecule predictions. The DBPP-Predictor standalone software is free and user-friendly including a convenient operator interface and an easy-to-understand result output for nonexperts in computer-aided drug design.

## Results and discussion

### Data set analysis

The approved drugs and non-drug compounds sampled from three databases (ZINC, ChEMBL and GDB17) were used for model training and testing. Table 1 summarized the number and composition of compounds in each data set. After the data preprocessing procedures, 5147 drugs were obtained, containing 2679 and 2468 compounds for the FDA-approved and the other region-approved, respectively. 10,000 molecules were sampled from each of the three databases as negative samples. The PU learning strategy was used for data noise analysis of negative sets. Additional file 1: Table S5 described the analysis results of the non-drug samples. The results indicated that the ChEMBL molecules had higher drug similarity compared to the other two databases. Meanwhile, three extra real-world sample sets (Investigation, WITHDRAWN and TCMSP) were prepared for model evaluation, consisting of 1751, 266 and 6574 molecules, separately. They were expected to bring valuable information for drug-likeness scoring evaluation. More details were available in the Additional file.

**Table 1:** Compound information in each data set

Type	Name	Composition
Training set	FDA_ZINC	2679 FDA + 2679 ZINC molecules
Test set	Worlddrug_ZINC	2468 Worlddrug + 7321 ZINC molecules
External validation sets	Worddrug_ChEMBL	2468 Worlddrug + 10,000 ChEMBL molecules
	Worddrug_GDB17	2468 Worlddrug + 10,000 GDB17 molecules
Others	Investigation	1751 Investigation group molecules
	WITHDRAWN	266 Withdrawn drugs
	TCMSP	6574 molecules from TCMSP database

**Fig. 2:**
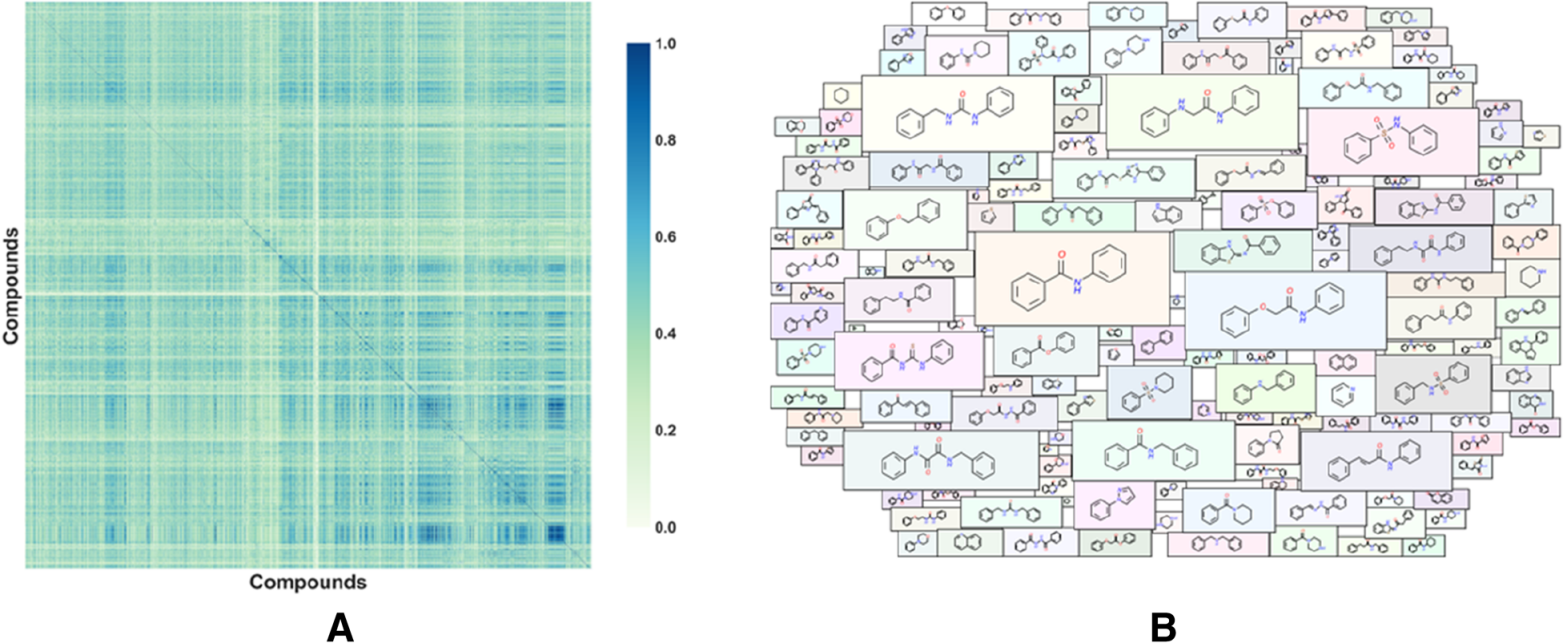
**A** Heat map of Tanimoto similarity of the total data set with MACCS fingerprint. **B** Molecular Cloud displayed the 150 most frequently occurring molecular scaffolds in the data set

To further explore the chemical space, we performed the principal component analysis (PCA), Tanimoto similarity analysis, and Murcko scaffold analysis on the comprehensive data set. It was obvious from PCA that the data had a wide distribution in space (Additional file 1: Fig. S1). Since the data sets overlapped relatively well, the reasonableness of the divided data should be recognized. The Tanimoto similarity index was calculated using MACCS and implemented with the RDKit package. As shown in Fig. 2A, the overall color of the Tanimoto similarity heat map was light green with an average similarity of 0.358, indicating that the structural diversity of the data set was clear. In addition, 3337 Murcko scaffolds were detected, with an average of 1.6 molecules contributing one new scaffold. More than 90% of Murcko scaffolds shared no more than 2 molecules, also demonstrating the high chemical diversity of the data set. We visualized the frequency of 150 scaffolds using a molecular cloud (Fig. 2B), where the scaffolds with a higher frequency of occurrence occupied a larger area.

### Analysis of property profiles

#### Property profiles and drug-likeness

The strategy for the prediction of chemical drug-likeness, namely DBPP-Predictor, was proposed on the basis of property profiles, which contained six physicochemical and 20 ADMET property endpoints. The ADMET property endpoints are binary classification models. The modeling data and model performance were shown in Additional file 1: Table S6. All models were built from over 500 high quality endpoint data. 70% of the models have a prediction accuracy of over 0.8. High-quality models are guaranteed for the property profiles. In addition, the correlation between the endpoints and the drug-likeness was analyzed, using the equation in Additional file 1: Text S1. As shown in Additional file 1: Fig. S2, the ADMET endpoints were ranked more highly. It indicated that the toxicity endpoints, like mutagenicity, oral acute toxicity, and genotoxicity, as well as transporter endpoints of the compound have a significant effect on drug-likeness.

#### Physicochemical property profile

**Fig. 3:**
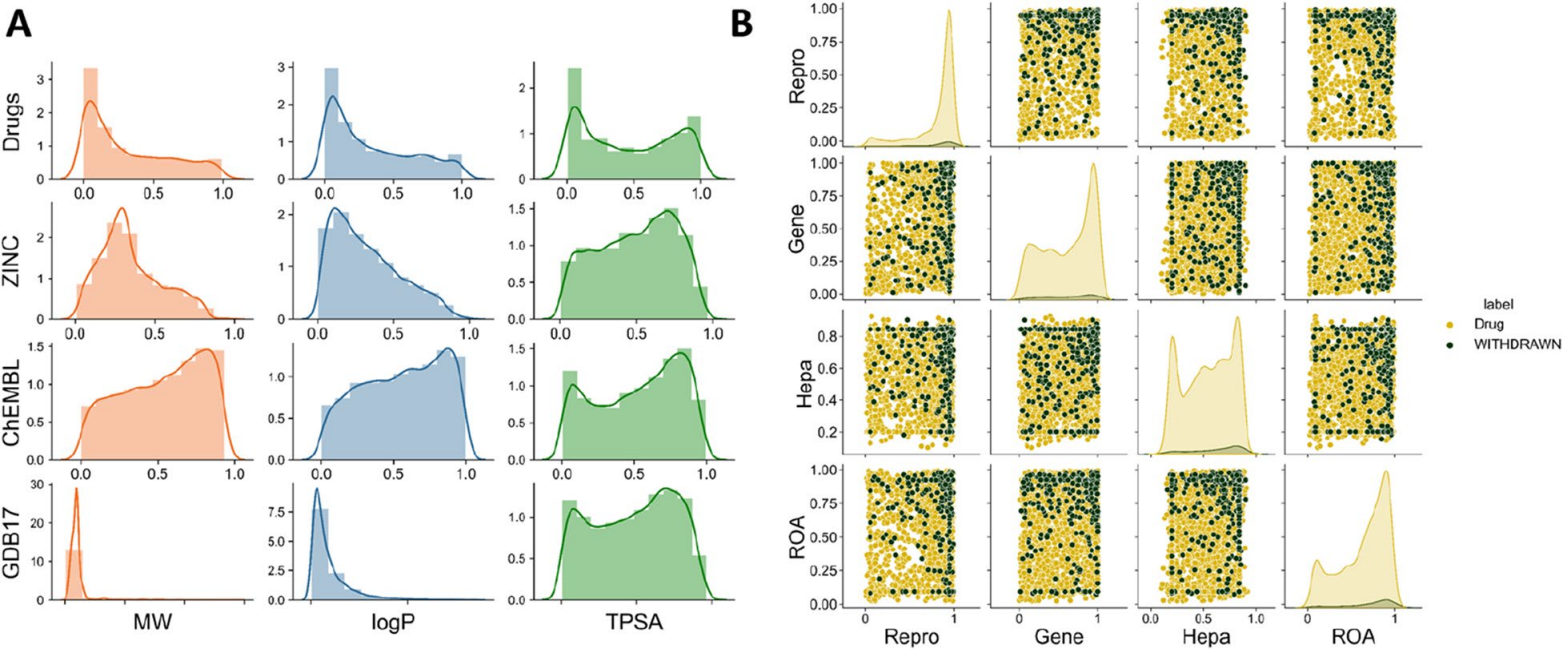
**A** Physicochemical property profiles of drugs and non-drugs (ZINC, ChEMBL and GDB17) correlation analysis, including MW, logP and TPSA. **B** Scatter matrix plot of the four toxicity endpoints analysis for drugs and withdrawals. Among them, Repro, Gene, Hepa, and ROA represent the property endpoints of respiratory toxicity, genotoxicity, hepatotoxicity and oral acute toxicity, respectively

The six physicochemical properties of Drugs, ZINC, ChEMBL, and GDB17 molecules were visualized. Figure 3A showed MW, logP and topological polar surface area (TPSA) probability density distributions of the compounds, which overlapped and prevented a clearcut separation. The distribution of the numbers of hydrogen bond acceptors (HBA), hydrogen bond acceptors (HBD) and rotatable bonds (nROT) were plotted in Additional file 1: Fig. S3. To discriminate drug-like compounds by one single property was considered too simple, and the quantitative assessment of drug-likeness using multiple parameters was a feasible approach. For example, based on seven physicochemical properties, the QED score was widely used in the assessment of generating drug-like molecules [16].

#### ADMET property profile

Efficacy and safety are two key characteristics for a compound to become a drug. Appropriate pharmacokinetic properties influence the drug efficacy. Therefore, ADMET properties are also used as property profile in DBPP-Predictor. Additional file 1: Fig. S4 presented the visualization result of the ADME property profile for drug and non-drug molecules. Among that, HIA and Caco-2 endpoints were utilized to assess the absorption properties. The absorption rate and extent of a compound affect its bioavailability. The transporters played significant roles in many processes of compound effects in vivo. Inhibition of transporter proteins may lead to accumulation of the drug and produce adverse effects. In the transporter-related inhibitor assessment, non-drug molecules were considered to have significantly higher inhibitory potential than drug molecules at 83.3% (5/6) of the endpoints. From the clearance results, drug molecules may have a more sustained in vivo effect. Concerning absorption properties, the two ones did not show significant differences. Compared to non-drug molecules, withdrawals had plausible pharmacokinetic properties. Adverse reactions and toxic effects were responsible for most of the withdrawals. The results of our analysis of drugs and withdrawals for toxicity corroborated this conclusion. Figure 3B demonstrated the correlation analysis of several toxicity endpoints of drugs and withdrawals, which revealed that most of the withdrawals had a higher propensity for toxicity, such as respiratory toxicity, genotoxicity, hepatotoxicity, and oral acute toxicity. Therefore, we believed that a comprehensive toxicity potential screening in the property profile was of great interest for drug-likeness prediction. However, it was also evident from the results that not all drugs have low toxicity scores. Actually, a successfully marketed drug was not necessarily the molecular candidate with perfect properties, while the balance of multiple properties required more attention.

Furthermore, the importance of each ADMET endpoint was analyzed with SHapley Additive exPlanations (SHAP) [44] to provide guidance for understanding DBPP-Predictor. SHAP was utilized for model interpretation through feature attribution. Additional file 1: Fig. S5 depicted the ADMET features important to all the investigation observations. The importance of a feature was obtained from the mean of absolute SHAP attributions. Details of the SHAP values were available in Additional file 1: Table S7. As seen in Additional file 1: Fig. S5, toxicity features drove the drug-likeness prediction down, including hepatotoxicity, mutagenicity, oral acute toxicity, genotoxicity, and carcinogenicity. It implied that the higher is the toxicity risk of a compound, the lower its drug-likeness is. The opposite was observed for oral bioavailability, which had a positive contribution. The mitochondrial membrane potential got the largest feature contribution in absolute SHAP value, without a significant linear relationship for drug-likeness contribution. 66.7% of the efflux transporter inhibitors had negative SHAP values, while OATP1B3 and OATP1B1 inhibitors had higher drug-likeness contributions.

### Performance of models

We utilized three traditional ML methods and four GNNs to build models for prediction of drug-likeness. Six different types of molecular representations were employed to evaluate the models. Grid search and Bayesian search were used for parameter optimization of traditional ML and GNN, respectively. The optimal parameters of the models were available in Additional file 1: Tables S8, S9. Feature normalization brought beneficial effects to the models, as shown in Additional file 1: Table S10.

#### Performance of ten-fold cross-validation

In this study, the hyperparameter $\upgamma$ was introduced to regulate the combined weight between the physicochemical property and the ADMET property profiles to optimize the DBPP-Predictor performance. From the results shown in Additional file 1: Fig. S6, it was apparent that the DBPP-Predictor benefited from the hybrid representation strategy. According to the AUC and F1 values, $\upgamma$ = 0.6 was selected as the optimal parameter. To assess the model performance, we conducted a comparative study with six different representations, involving classical ML and DL algorithms. Table 2 depicted the performance of the models coupled with different representations on the ten-fold cross-validation. Optimal models based on different representations were selected for further testing. The ten-fold validation results for all models were available in Additional file 1: Table S11. By comparing the molecular representations, it could be found that all models had considerable abilities to distinguish drugs from non-drugs in the training sets, excluding the single QED-based one. The model based on QED representation performed from 59.1% to 68.4% for the five indicators in ten-fold cross-validation. The cross-validation accuracy, recall and SP values of the other four models ranged from 0.901 to 0.984, 0.899 to 0.984, and 0.818 to 0.992, respectively. The AUC values were typically evaluated for the performance of binary classification. The ADMET property-based model got the best AUC value, yielding AUC 0.996 in internal validation.

**Table 2:** Ten-fold cross-validation results for models based on different representation

Representation	Accuracy	Precision	Recall	AUC	SP
Descriptors	0.968 ± 0.001	0.965 ± 0.003	0.972 ± 0.001	0.994 ± 0.000	0.964 ± 0.003
FP	0.972 ± 0.002	0.976 ± 0.002	0.967 ± 0.002	0.994 ± 0.000	0.976 ± 0.002
GCN	0.901 ± 0.045	0.849 ± 0.074	0.984 ± 0.016	0.988 ± 0.002	0.818 ± 0.104
QED	0.627 ± 0.004	0.637 ± 0.005	0.591 ± 0.001	0.684 ± 0.002	0.663 ± 0.007
ADMET Property	0.984 ± 0.000	0.992 ± 0.000	0.975 ± 0.000	0.996 ± 0.000	0.992 ± 0.000
Property Profiles	0.903 ± 0.001	0.905 ± 0.002	0.899 ± 0.003	0.961 ± 0.001	0.906 ± 0.002

#### Evaluation of the test set and external validation sets

**Fig. 4:**
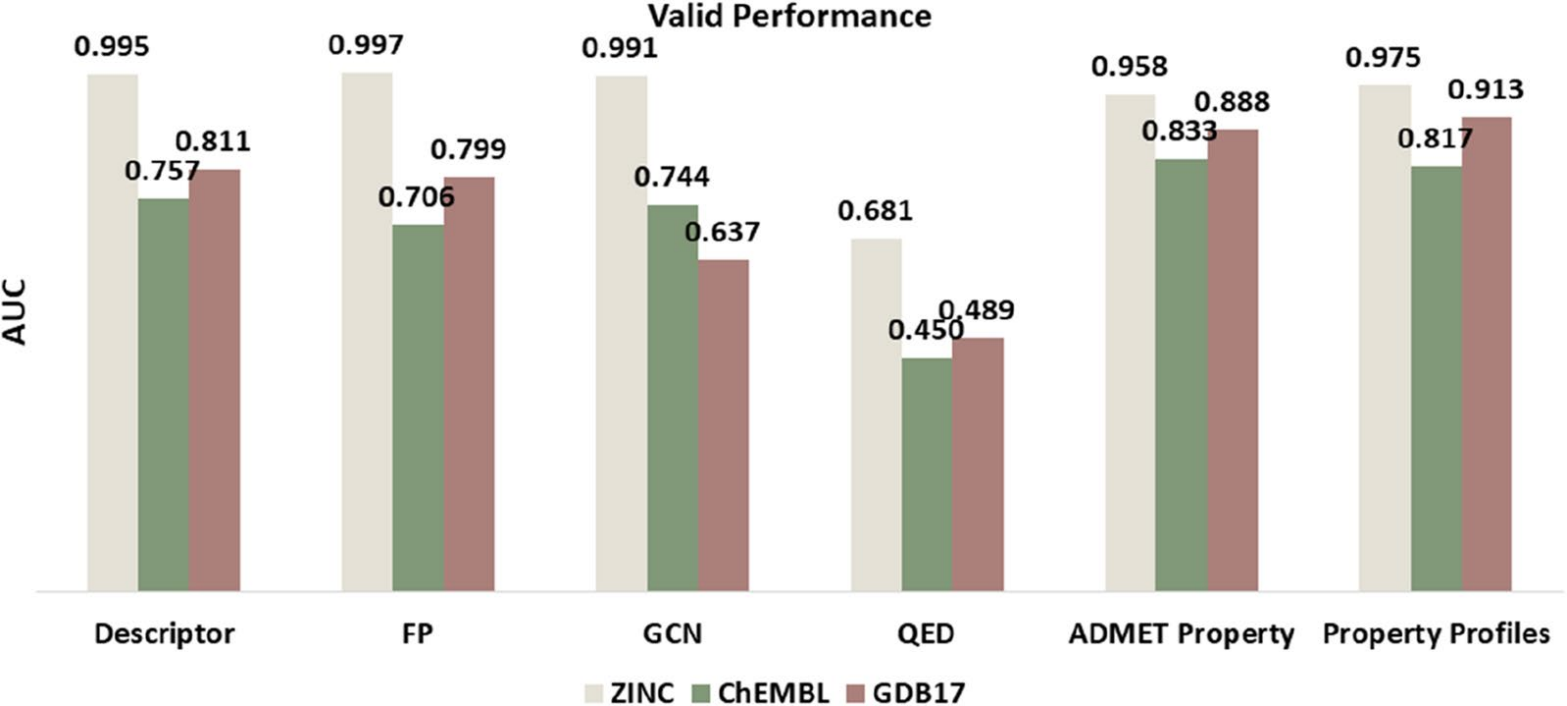
Model performance on external validation sets with different representations

Although the models achieved satisfactory prediction performance in cross-validation, it was necessary to further explore the generalization performance of the models. Therefore, we evaluated the models using test set and external validation sets. For the test set (Worlddrug_ZINC), the results showed that each model still achieved good performance, consistent with ten-fold cross-validation (Additional file 1: Table S12). Comparing the AUC values (Fig. 4), the descriptor-based and fingerprint-based approaches outperformed the DBPP-Predictor model. The reason for that might be related to molecular similarity. In addition, the GCN model showed an AUC of 0.991, which was promising. For the Worlddrug_ChEMBL and Worlddrug_GDB17 external validation sets, all models performed worse, decreasing from 13.0% to 35.5%. The GCN model performed the worst, with AUC 0.637 in the Worlddrug_GDB17 set. The generalization ability of the DL models was strongly affected by the size of the training data. Certainly, considering the performance of the combination of the three external validation sets, the DBPP-Predictor model demonstrated stronger robustness and better generalization. It yielded an average AUC value of 0.902, which was 14.0% improved compared to the GCN model (average AUC = 0.791). The results indicated that the ability of QED to discriminate between drug and non-drug-like compounds was indeed overestimated. The models based on QED representation displayed performance with AUC values below 0.5.

#### Analysis of sample dependence

**Fig. 5:**
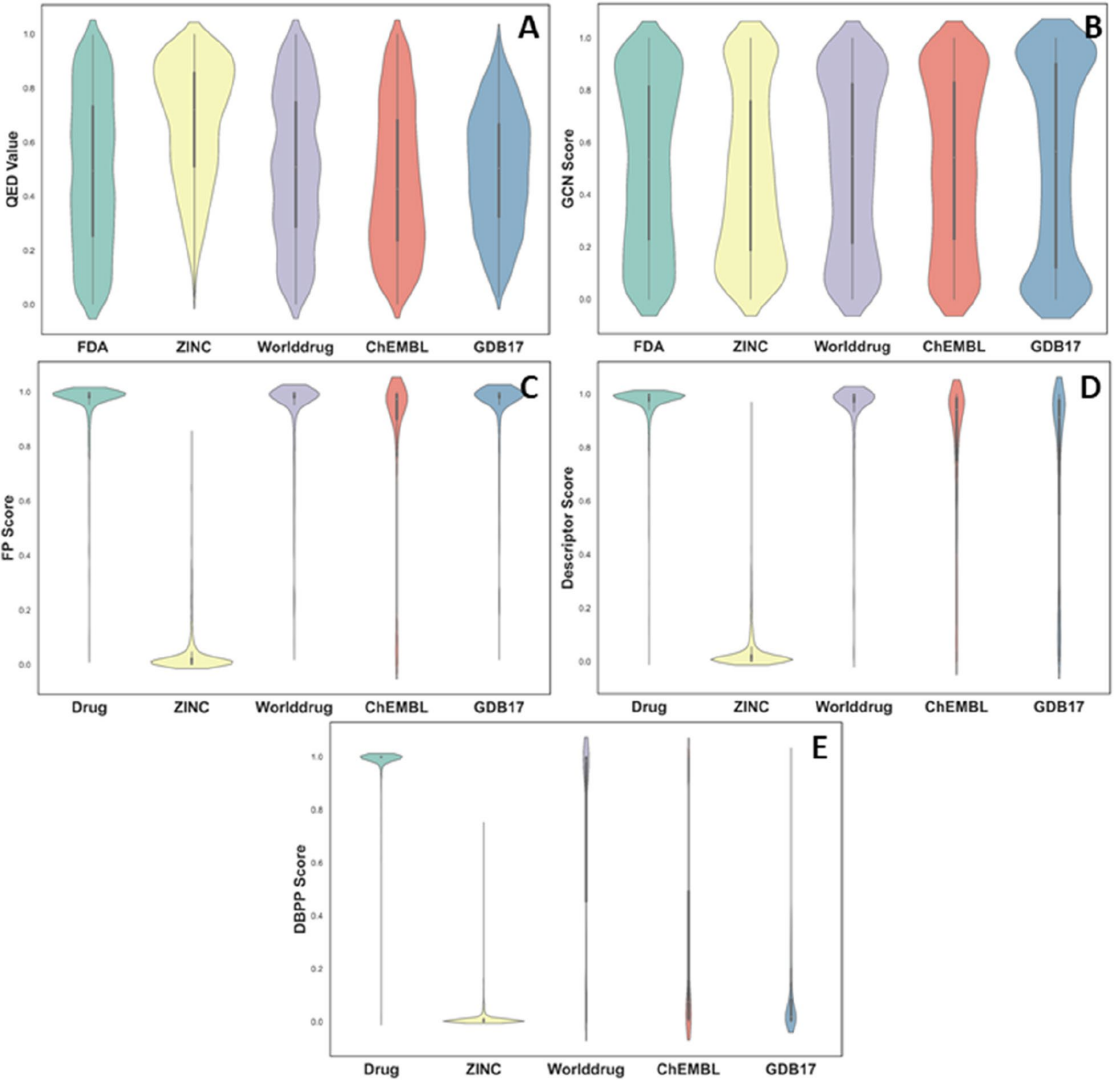
Drug-likeness scoring violin plots for five data sets and analysis of sample dependence. **A** QED scores. **B** GCN scores. **C** FP scores. **D** Descriptor scores. **E** DBPP scores

The decreased model performance was found in external validation sets with independent negative samples from ChEMBL and GDB17, hence different models were explored for sample dependence. Figure 5 depicted the generalization ability of the models in different negative samples, where QED was used for comparison. The QED values failed to discriminate drugs from non-drugs, as shown in Fig. 5A. The possible reason of high QED values on ZINC samples might be because they were made to obey Ro5. The QED values were reported to have a poor ability to distinguish between drugs and non-drugs [18, 19]. The GCN model was unsatisfactory with poor generalization ability, as reflected in Fig. 5B. Without enough data, the deep neural networks did not learn task-relevant knowledge well. Adequate data support was required for complex network parameters. Introduction of transfer learning and data augmentation strategies would be beneficial. The performance of the models based on molecular fingerprints and descriptors were shown in Fig. 5C and D. It was apparent that the two types of representation were overly dependent on training data. The features from training data, FDA drug set and ZINC non-drug set, were learned for discriminating between positive and negative samples. However, these models had unsatisfactory generalization performance. It was debatable that most compounds in ChEMBL and GDB17 were scored with high drug-likeness. The results indicated that structure-based representations (fingerprints and descriptors) were not good enough for drug-likeness prediction. The scoring of DBPP-Predictor (DBPP score) was shown in Fig. 5E. It could be seen that the DBPP-Predictor models distinguished the training data very well, while sensible for data from ChEMBL and GDB17. The mean scores for the ChEMBL and GDB17 sets were 0.276 and 0.105, respectively. Therefore, it was believed that the DBPP score would have good generalization ability and promising applications.

### DBPP-Predictor scoring feasibility

The DBPP-Predictor framework has several advantages over previous method. Compared with experimental methods, DBPP-Predictor was rapid and efficient for drug-likeness assessment and provided guidance for drug development. Meanwhile, it was packaged as a standalone software, facilitating user-friendly drug-likeness prediction and information protection of compounds. DBPP-Predictor showed good predictive performance and better generalizability on test sets and external validation sets compared with others. It also demonstrated considerable plausibility and feasibility in evaluation of various data sets.

#### Plausible evaluation of real-world samples

**Fig. 6:**
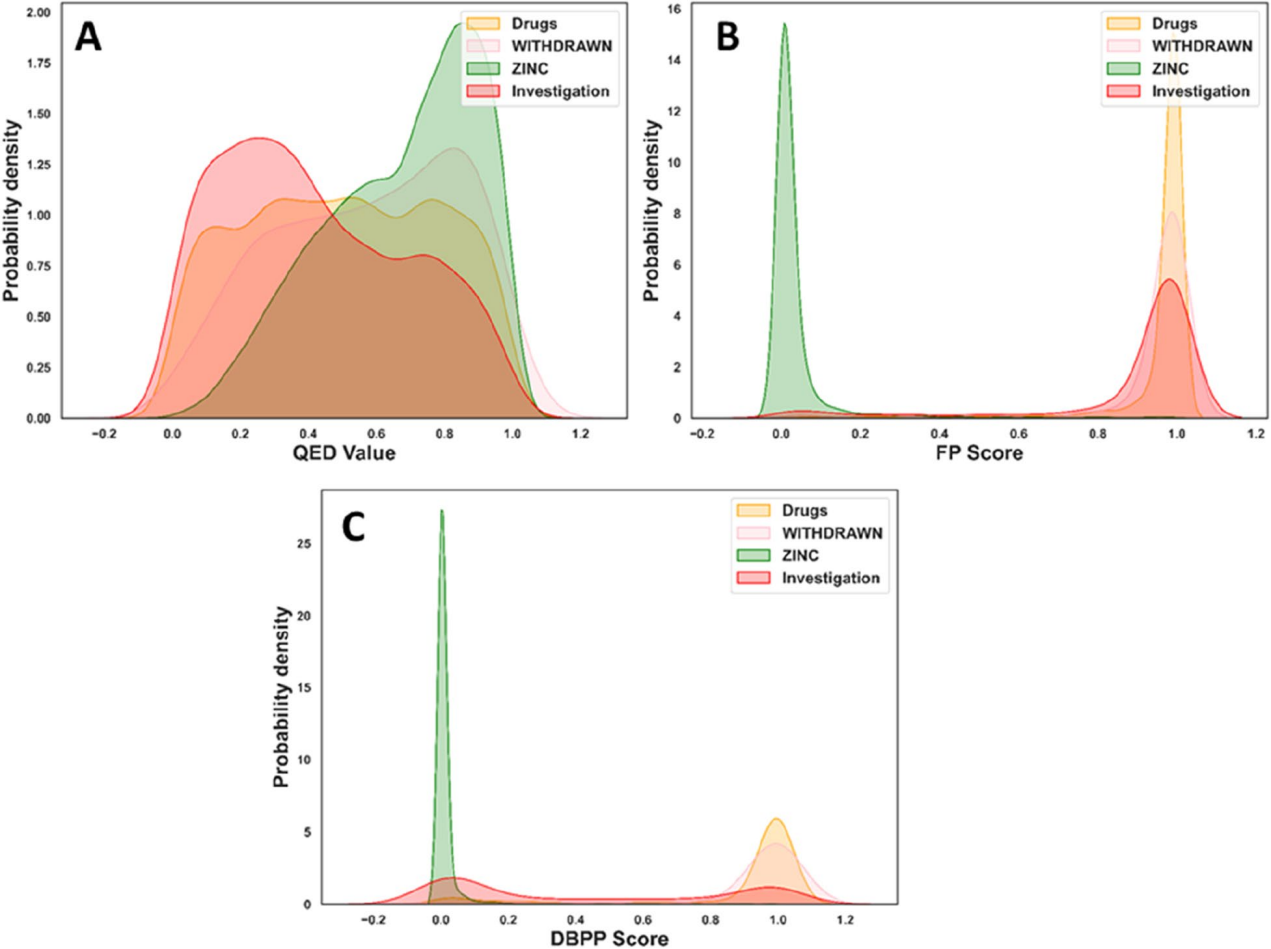
Comparison of **A** QED value, **B** FP score and **C** DBPP score on real-world sets

Here, 266 withdrawn drugs and 1751 compounds from the DrugBank investigation group were used separately. They were used to test the feasibility of models in assessment of real-world samples. The output of the classification models was interpreted as the probability that a query compound had the desired drug-likeness. The QED value corresponding to the query compound was treated as the baseline. The QED values, fingerprint-based model scoring (FP score) and DBPP-Predictor scoring (DBPP score) for these data sets were shown in Fig. 6. The QED values for the Drugs, ZINC, Investigation and WITHDRAWN sets were 0.499, 0.673, 0.419 and 0.576, respectively. It was obvious that QED value could not distinguish the four data sets (Fig. 6A). As shown in Fig. 6B, the scores of the fingerprint-based model could discriminate the four data sets, from the ZINC set with the lowest score to Drugs set with the highest score. However, the FP scores still failed to discriminate the drugs from the investigation compounds and withdrawn drugs. In comparison, DBPP-Predictor gave a more reasonable scoring distribution for these data sets as shown in Fig. 6C. The DBPP score can clearly distinguish between the Drugs set and the ZINC set. DBPP-predictor scored the drug candidates realistically, with a score of 0.428. Approximately 90% of drug candidates were reported to fail in clinical testing [45], while only a few compounds would be approved for marketing. For the withdrawn drugs, DBPP-Predictor still tended to give them high drug-likeness scores like the drugs on sale. It meant that DBPP-Predictor was unable to make a satisfactory distinction between the two sets. The outcome was intelligible. Drug-likeness is not an intrinsic property of a compound [46]. The marketing or withdrawal of a drug will be influenced by a complex consideration of numerous factors [47–49].

#### Screening assessment of databases

To test the feasibility of DBPP-Predictor scoring, the average DBPP scores of the five data sets were calculated. As shown in Table 3, the drug and non-drug data sets were scored from high to low. Meanwhile, the Mann–Whitney U test was applied to calculate the differences in DBPP scores between the data sets. The results suggested that DBPP score could significantly distinguish among data sets (Additional file 1: Table S13) and be considered as a good indicator for drug-likeness assessment. The DBPP score of the Worlddrug set (0.736) was adopted as the threshold for drug-likeness. A compound with a DBPP score greater than the threshold was recommended as druggable. For the 6574 natural products in the TCMSP set, DBPP-Predictor gave a higher score (0.801) compared to ZINC, ChEMBL and GDB17. It was plausible because natural products were important sources of drugs. In addition, natural-product-inspired synthetic compounds also provided viable and innovative solutions to drug discovery. Meanwhile, the property profile visualization was available in DBPP-Predictor. Researchers can conveniently obtain the drug-likeness score of a query molecule while obtaining its visual property information. Drug-likeness scoring and image-based property information could be easily obtained from DBPP-Predictor. Researchers can modify and optimize unsatisfactory properties to get the ideal molecules.

**Table 3:** DBPP scores on various data sets

Name	Worlddrug	TCMSP	ZINC	ChEMBL	GDB17
Number	2468	6574	7321	9954	10,000
DBPP Score	0.736	0.801	0.018	0.277	0.101

#### Comparison with other scores

**Fig. 7:**
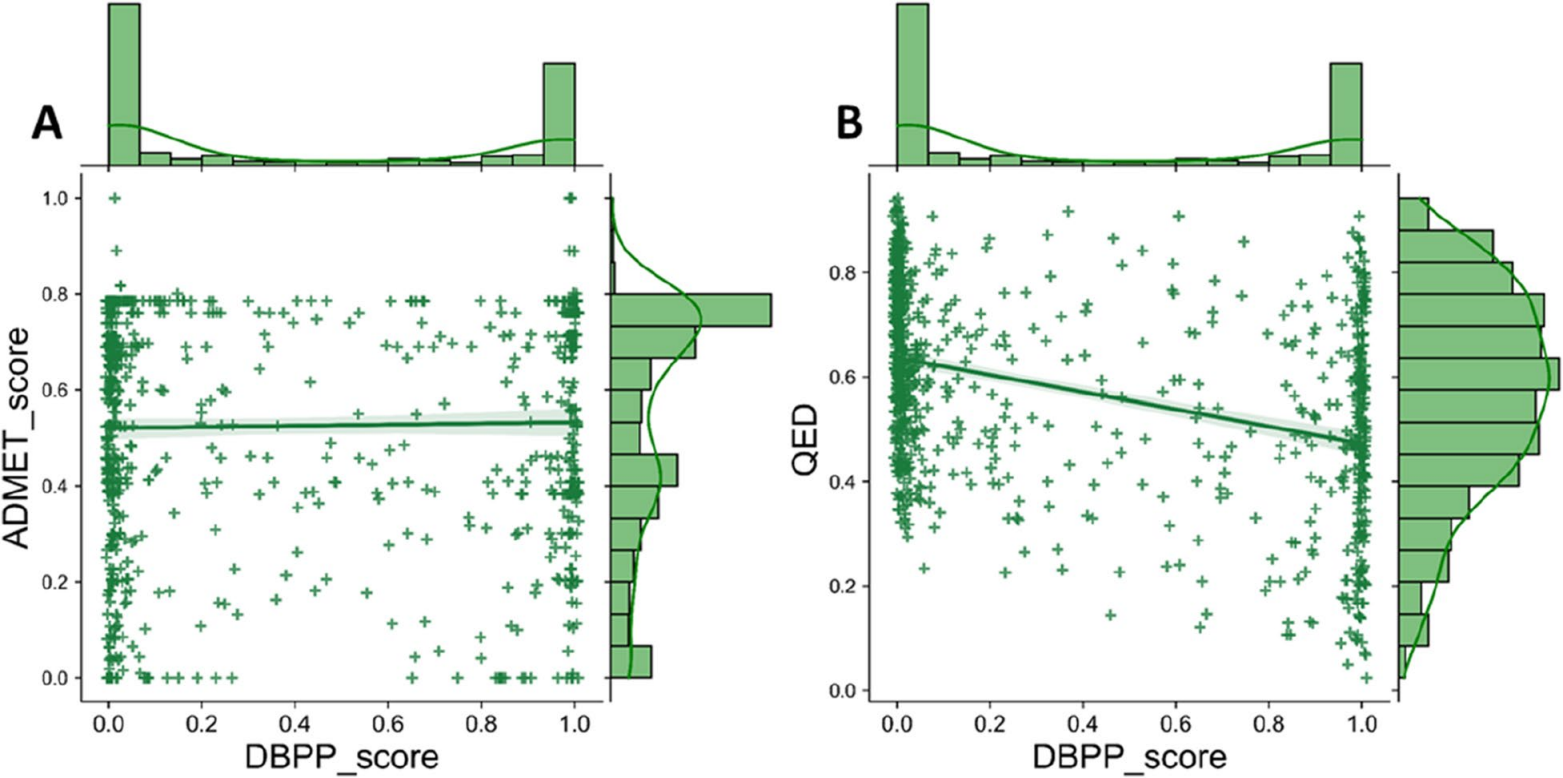
Correlation of drug-likeness evaluation between **A** ADMET-score and DBPP score, **B** QED and DBPP score

We further compared DBPP score with QED value and ADMET-score [17] by a comprehensive data set consisting of data from different sources. The details of this data set were presented in Additional file 1: Table S14**.** As shown in Fig. 7, a low linear correlation coefficient was found between all three scores. From the scoring distribution, it could be noticed that the DBPP score had different concerns from QED value and ADMET-score. The DBPP score was designed to provide a judgment reference for the drug-likeness of the compounds. By combining the drug-likeness threshold (0.736), we found that 286 compounds out of the 800 compounds to be tested had good drug-likeness, notably containing 200 known drugs. We considered that the druggable assessment of the DBPP score was efficient and reasonable. It captured known drugs efficiently and was able to explore potential druggable molecules in chemical space. The QED value and ADMET-score, on the other hand, relied on oral drug data at the time of the study to explore drug-like compounds in terms of physicochemical and ADMET properties, respectively. The scoring results demonstrated that known drug and non-drug molecules received QED scores of 0.539 and 0.622, respectively. The ADMET-score yielded mean scores of 0.548 and 0.519 for drugs and non-drugs, separately. Neither of them performed well in advising whether a query molecule would be a drug or not, but their values for molecular property assessment were undeniable. There might be a tendency to give more attention to compounds with higher QED values because they may have better physicochemical properties.

### Case study

**Fig. 8:**
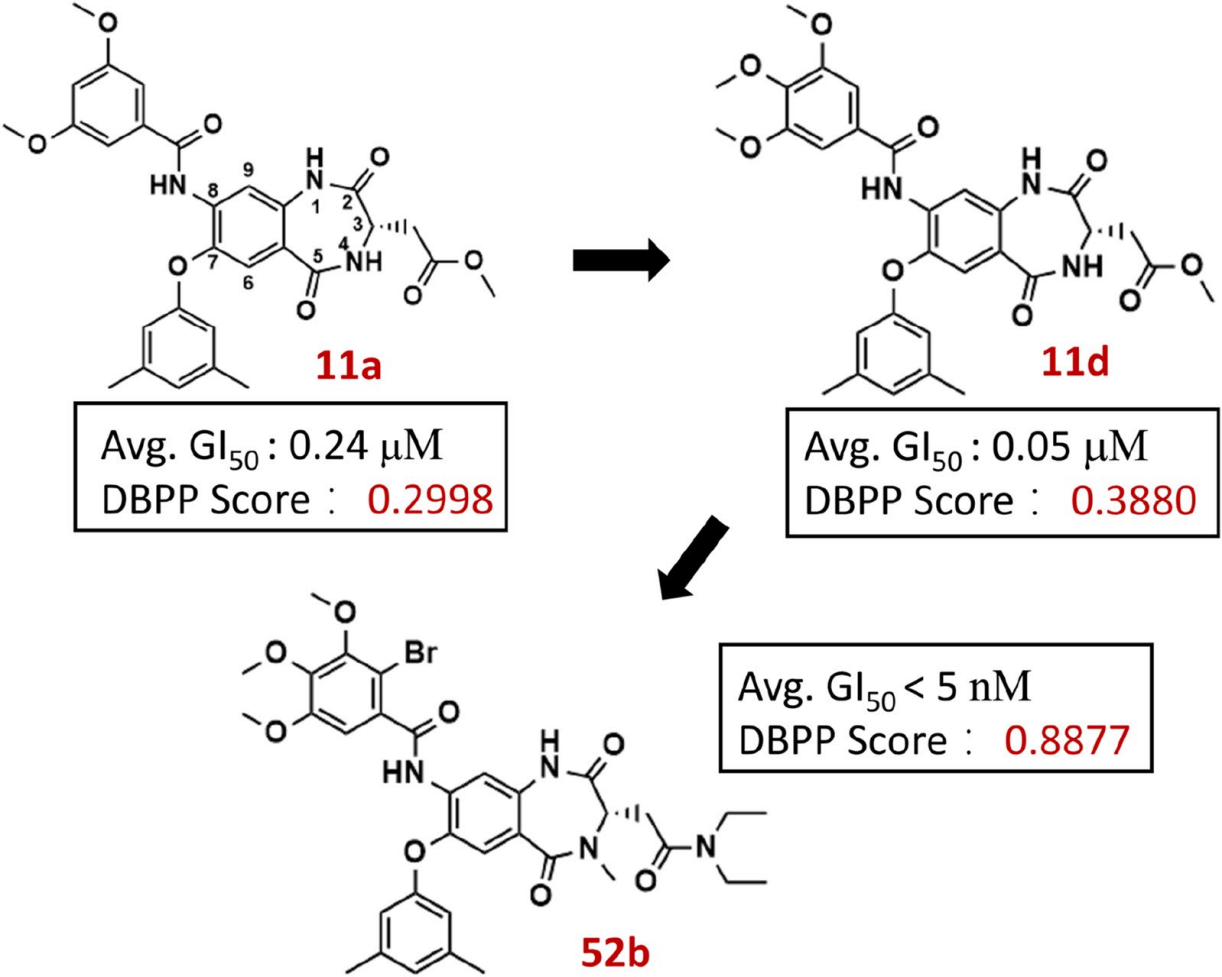
The average 50% growth inhibitory concentration (Avg. GI_50_) and DBPP scores of the designed compounds (**11a**, **11d** and **52b**). Avg. GI_50_ represents the average GI_50_ value against 60 human cancer cell lines

1,4-benzodiazepine-2,5-dione (BZD) derivatives were found to exhibit multiple antitumor cell growth activities in vitro [50]. The initial hit compound (**11a**) was further modified. After systematic optimization and SAR studies, a new class of BZD derivatives represented by compound **52b**, was reported. They all exhibited efficient anticancer activities in vitro and were promising as efficient potential inhibitors of protein synthesis. We performed DBPP-Predictor on each of the 52 molecules synthesized in this study. Details of DBPP scores were available in Additional file 1: Table S15 and Additional file 2: Table S16. The drug-likeness predictive values of the molecules were mapped to the GI_50_ experimental values. Figure 8 illustrated some representative examples. The results showed that our DBPP-Predictor successfully predicted the trend of optimization in the study, consistent with the experimental results. Details were shown as follows.

Modification of **11a** at C8-position. Furan-2 carbonyl (**11b**) or 4-fluorobenzoyl (**11c**) were found to not show anticancer activity. While 3,4,5-trimethoxybenzamide (**11d**) was more potent than the hit compound **11a**. From the DBPP-Predictor, the drug-likeness scores of **11a**, **11b**, **11c**, and **11d** were 0.2998, 0.0118, 0.2074, and 0.3880, respectively. DBPP-Predictor successfully predicted the directionality of changes in anticancer activity, consistent with experimental reports.

Optimization with** 11d **as Hit. Carried out with the potential metabolic instability problem present in **11d**, optimizing its pharmacokinetic properties. Impressively, **21d**, substituted with diethyl amide at the R3 position, displayed two-fold higher cellular potency than **11d**. Compound **21c** also showed decent improvement. Their average GI_50_ values were 0.03 μM and 0.06 μM, respectively. The scores given by DBPP-Predictor were 0.5770 for **21c** and 0.6775 for **21d** with significant improvements compared to **11d**. The DBPP scores also reflected the experimental results that **21d** had better cellular potency than **21c**. To reduce the polar surface area and improve lipophilicity, the three polar amide groups were methylated, yielding compounds **36**, **37**, and **34a**. The experimental results showed a decrease of compounds **36** and **37** and an increase of compound **34a** in cellular potency. The DBPP scores corroborated the experimental results, giving scores of 0.5633, 0.4947 and 0.8598 for compounds **36**, **37**, and **34a**, respectively.

The optimization trend from** 11a** to **52b**. Drug-likeness scores of 0.1864 and 0.8512 were obtained for the series **11** (**11a**-**11r**) and series **52** (**52a**-**52i**) compounds, respectively. The series **52** compounds were a new class of BZD with different halogenated substituents, represented by **52b** (DBPP score = 0.8877). The introduction of halogenated substituents improved the hydrophobicity and transmembrane permeability of the compounds. They had better pharmacokinetic properties, such as hydrophobicity and transmembrane permeability.

### Generalizability and interpretability of DBPP-Predictor

DBPP-Predictor demonstrates robustness across various scales and characteristics in external validation. It provides reasonable drug-likeness scores for individual compounds and databases. The discriminative ability of DBPP-Predictor between drug-like and non-drug-like compounds, along with its feasibility for real-world sample assessment, is noteworthy. Moreover, the interpretable representation enhances the credibility and value of drug-likeness assessment. Within DBPP-Predictor, compounds are characterized by 26 attributable properties closely related to drug-likeness. Researchers can utilize these accessible property profiles to strategically optimize and modify target compounds, thereby improving their drug-likeness. Despite the aforementioned advantages, there is still room to improve our method. DBPP-Predictor, based on binary classification data, acknowledges the inherent potential bias in the training data. Furthermore, understanding the limitations of the 26 selected properties as molecular representation in this study is crucial for leveraging DBPP-Predictor in drug-likeness assessment and method refinement.

### Interface and functions of the standalone software

**Fig. 9:**
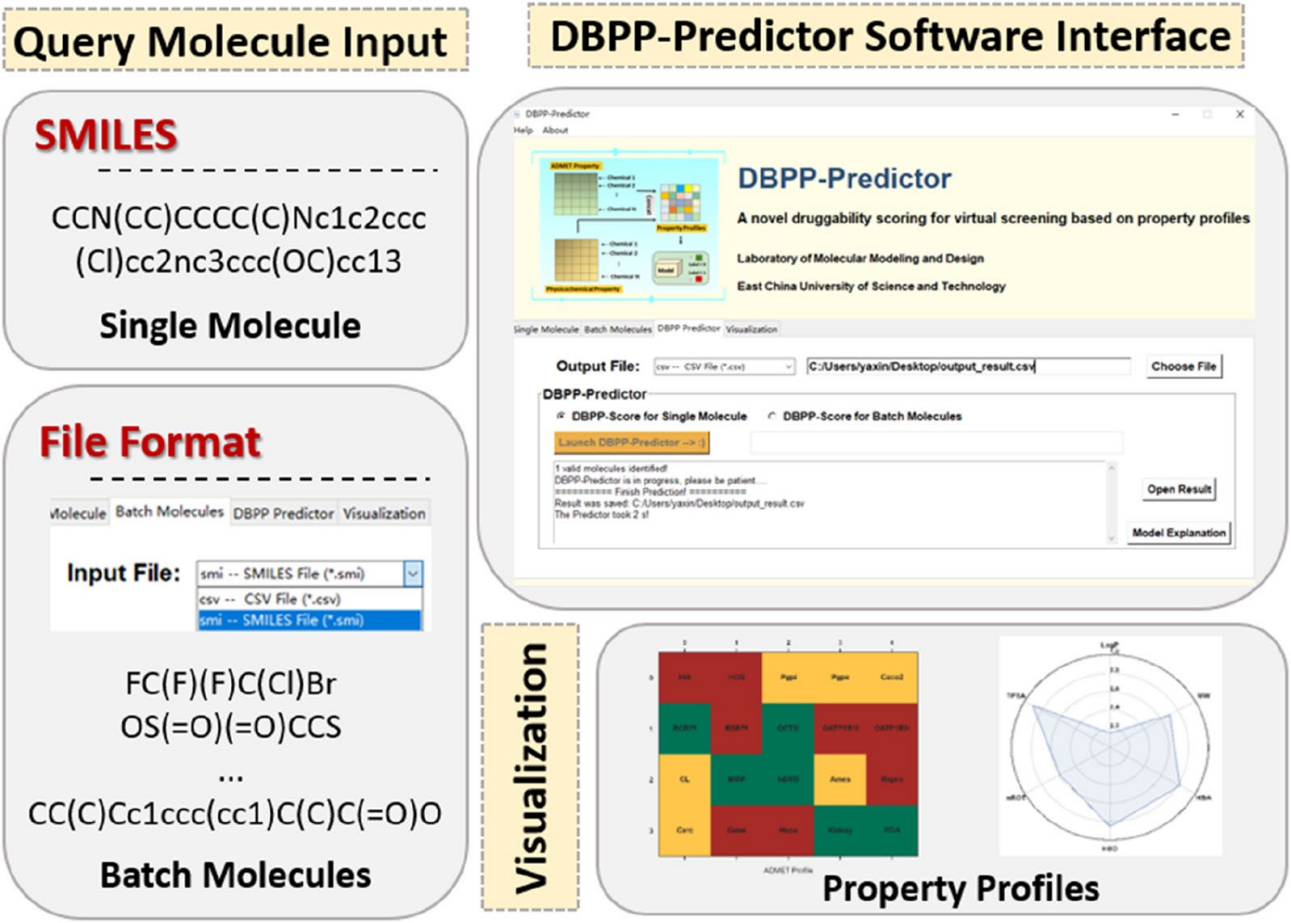
The interface of the standalone software DBPP-Predictor. Two options are available for users to predict drug-likeness assessment of single molecule or batch molecules. Visualization function provides easy-to-understand interpretation of the property profiles

The interface and functions of the standalone software DBPP-Predictor was displayed in Fig. 9. Two types of prediction, namely *Single Molecule* and *Batch Molecules*, are available to support drug-likeness prediction for single molecule and batch molecules. The query molecule should be represented as a canonical SMILES string, with input checks before prediction. Then, users can select the output path of the prediction results and click *Launch DBPP-Predictor* to start the prediction. The prediction results will be stored in CSV format in the selected output path. To facilitate understanding, the result interpretation file is conveniently available. In addition, DBPP-Predictor provides a visualization module for property profiles. If users are unsatisfied with the drug-likeness score of the molecule and would like to conduct optimization study, we recommend to use the visualization module. The property profile information of the target molecule will provide the user with optimization guidance.

## Conclusions

We developed a novel scoring function, namely DBPP-Predictor, for the prediction of chemical drug-likeness based on hybrid property profile representation, integrating physicochemical and ADMET properties. Compared with other representations, the property profile-based models achieved better performance on the test sets and external validation sets, which demonstrated its potential for drug-likeness assessment. Moreover, relatively low sample dependence was observed in DBPP-Predictor. With the evaluation of various data sets and the case study in compound optimization, DBPP-Predictor demonstrated the feasibility of application in drug screening and optimization. In addition, a free user-friendly standalone software was developed to facilitate drug-likeness assessment and property visualization. We believe that DBPP-Predictor would become a valuable tool for the prediction of chemical drug-likeness in drug discovery and development.

### Supplementary Information

**Additional file 1: Table S1.** The definition of initial canonical atom feature. **Table S2.** The definition of initial canonical bond feature. **Table S3.** The definition of initial AttentiveFP atom feature. **Table S4.** The definition of initial AttentiveFP bond feature. **Table S5.** The PU learning analysis results of non-drug samples. **Table S6.** Data details and model performance of the ADMET endpoints. **Table S7.** The SHAP value analysis for the ADMET endpoints. **Table S8.** Traditional machine learning model parameters. **Table S9.** Graph neural network model parameters. **Table S10.** Impact of feature normalization on the model. **Table S11.** The ten-fold cross-validation results for all models. **Table S12.** The test set results for all models. **Table S13.** P values of DBPP predictor on various data sets. **Table S14.** Data set information of 800 data for score analysis. **Table S15.** DBPP scores of the 52 molecules in case study. **Text S1.** The equation of correlation analysis. **Figure S1.** Three-Dimensional principal component analysis on the training, test and validation set. **Figure S2.** Heat map of property profiles endpoints and drug-likeness correlation analysis. **Figure S3.** PC property profiles distplot figure of drugs and nondrugs correlation analysis. **Figure S4.** ADME property profiles barplot figure of drugs and nondrugs correlation analysis. **Figure S5.** Analysis of the SHAP values for ADMET endpoints. **Figure S6.** The performance of DBPP model corresponding to different values of γ.

**Additional file 2: Table S16.** Details of the case study results.

## Data Availability

The DBPP-Predictor standalone software, source code and data sets and used in this article can be found at https://github.com/yxgu2353/DBPP-Predictor. The software tools, including RDKit (http://www.rdkit.org), Scikit-learn (https://scikit-learn.org/), DGL (https://www.dgl.ai/), LightGBM (https://github.com/microsoft/LightGBM), DescriptaStorus (https://github.com/bp-kelley/descriptastorus) and PyTorch (https://pytorch.org/) are freely available at their websites.

## Abbreviations

ADMET: Absorption, distribution, metabolism, excretion and toxicity
AUC: Area under the curve
AtomPairs: AtomPairs fingerprint
AttentiveFP: AttentiveFP network
BZD: 1,4-Benzodiazepine-2,5-dione
DL: Deep learning
ECFPs: Extended connectivity fingerprints
FPR: False positive rate
GNN: Graph neural network
GCN: Graph convolutional network
GAT: Graph attention network
GraphSAGE: Graph sample and aggregate network
HBA: Number of hydrogen bond acceptors
HBD: Number of hydrogen bond acceptors
LR: Logistic regression
ML: Machine learning
MACCS: MACCS fingerprint
Morgan: Morgan fingerprint
MW: Molecular weight
nROT: Number of rotatable bonds
PU learning: Positive unlabeled learning
PCA: Principal component analysis
QED: Quantitative estimate of drug-likeness
Ro5: Rule-of-five
RDKFingerprint: RDK fingerprint
ROC: Receiver operating characteristic
ROA: Oral acute toxicity
SVM: Support vector machine
SP: Specificity
SHAP: SHapley Additive exPlanations
TopoTorsion: TopoTorsion fingerprint
TPR: True prediction rate
TPSA: Topological polar surface area
WDI: World Drug Index
