# Supplementary material for: DBPP-Predictor: a novel strategy for prediction of chemical drug-likeness based on property profiles
Source: J Cheminform. 2024 Jan 5;16:4. doi: 10.1186/s13321-024-00800-9 (PMC10771006; doi:10.1186/s13321-024-00800-9)
Supplement: Supplementary file 1 — Additional file 1: Table S1. The definition of initial canonical atom feature. Table S2. The definition of initial canonical bond feature. Table S3. The definition of initial AttentiveFP atom feature. Table S4. The definition of initial AttentiveFP bond feature. Table S5. The PU learning analysis results of non-drug samples. Table S6. Data details and model performance of the ADMET endpoints. Table S7. The SHAP value analysis for the ADMET endpoints. Table S8. Traditional machine learning model parameters. Table S9. Graph neural network model parameters. Table S10. Impact of feature normalization on the model. Table S11. The ten-fold cross-validation results for all models. Table S12. The test set results for all models. Table S13. P values of DBPP predictor on various data sets. Table S14. Data set information of 800 data for score analysis. Table S15. DBPP scores of the 52 molecules in case study. Text S1. The equation of correlation analysis. Figure S1. Three-Dimensional principal component analysis on the training, test and validation set. Figure S2. Heat map of property profiles endpoints and drug-likeness correlation analysis. Figure S3. PC property profiles distplot figure of drugs and nondrugs correlation analysis. Figure S4. ADME property profiles barplot figure of drugs and nondrugs correlation analysis. Figure S5. Analysis of the SHAP values for ADMET endpoints. Figure S6. The performance of DBPP model corresponding to different values of γ. [file 13321_2024_800_MOESM1_ESM.docx]

**Additional file**

**DBPP-Predictor: a novel strategy for prediction of chemical drug-likeness based on property profiles**

Yaxin Gu, Yimeng Wang, Keyun Zhu, Weihua Li, Guixia Liu, Yun Tang*

Shanghai Frontiers Science Center of Optogenetic Techniques for Cell Metabolism, Shanghai Key Laboratory of New Drug Design, School of Pharmacy, East China University of Science and Technology, Shanghai 200237, China

* To whom correspondence should be addressed.

Tel: +86-21-64251052

Fax: +86-21-64251033

Email: ytang234@ecust.edu.cn

**List of Tables**

**Table S1.** The definition of initial canonical atom feature.

**Table S2.** The definition of initial canonical bond feature.

**Table S3.** The definition of initial AttentiveFP atom feature.

**Table S4.** The definition of initial AttentiveFP bond feature.

**Table S5.** The PU learning analysis results of non-drug samples.

**Table S6.** Data details and model performance of the ADMET endpoints.

**Table S7.** The SHAP value analysis for the ADMET endpoints.

**Table S8.** Traditional machine learning model parameters.

**Table S9.** Graph neural network model parameters.

**Table 10.** Impact of feature normalization on the model.

**Table S11.** The ten-fold cross-validation results for all models.

**Table S12.** The test set results for all models.

**Table S13.** P values of DBPP predictor on various data sets.

**Table S14.** Data set information of 800 data for score analysis.

**Table S15.** DBPP scores of the 52 molecules in case study.

**Table S16.** Details of the case study results.

**List of Text**

**Text S1.** The equation of correlation analysis.

**List of Figures**

**Figure S1.** Three-Dimensional principal component analysis on the training, test and validation set.

**Figure S2.** Heat map of property profiles endpoints and drug-likeness correlation analysis.

**Figure S3.** PC property profiles distplot figure of drugs and nondrugs correlation analysis.

**Figure S4.** ADME property profiles barplot figure of drugs and nondrugs correlation analysis.

**Figure S5.** Analysis of the SHAP values for ADMET endpoints.

**Figure S6.** The performance of DBPP model corresponding to different values of γ.

**Table S1.** The definition of initial canonical atom feature.

| **Features** | **Descriptions** | |
| --- | --- | --- |
| Atom type | | One hot vector specifying the type of this atom: ['C', 'N', 'O', 'S', 'F', 'Si', 'P', 'Cl', 'Br', 'Mg', 'Na', 'Ca', 'Fe', 'As', 'Al', 'I', 'B', 'V', 'K', 'Tl',  'Yb', 'Sb', 'Sn', 'Ag', 'Pd', 'Co', 'Se', 'Ti', 'Zn',  'H', 'Li', 'Ge', 'Cu', 'Au', 'Ni', 'Cd', 'In', 'Mn', 'Zr',  'Cr', 'Pt', 'Hg', 'Pb'] |
| Hybridization | | sp, sp^2^, sp^3^, sp^3^d, or sp^3^d^2^. |
| Formal charge | | Integer electronic charge assigned to atom. |
| # Hs | | Several bonded Hydrogen atom. (0-4) |
| Degree | | 0-10 |
| Implicit valence | | 0-6 |
| In aromatic ring | | Whether the atom is aromatic |

**Table S2.** The definition of initial canonical bond feature.

| **Features** | **Descriptions** |
| --- | --- |
| Conjugated | Whether the bond is conjugated. |
| In ring | Whether the bond is part of a ring. |
| Bond type | Single, double, triple, or aromatic. |
| Stereo | ‘Stereonone’, ‘Stereoany’, ‘Stereoz’, ‘Stereoe’,’Stereocis’, ‘Stereotrans’. |

**Table S3.** The definition of initial AttentiveFP atom feature.

| **Features** | **Descriptions** | |
| --- | --- | --- |
| Atom type | | One hot vector specifying the type of this atom: ['B’, ‘C’, ‘N’, ‘O’, ‘F’, ‘Si’, ‘P’, ‘S’, ‘Cl’, ‘As’, ‘Se’, ‘Br’, ‘Te’, ‘I’, ‘At’, and ‘other’.] |
| Hybridization | | sp, sp^2^, sp^3^, sp^3^d, or sp^3^d^2^. |
| Formal charge | | Integer electronic charge assigned to atom. |
| # Hs | | Several bonded Hydrogen atom. (0-4) |
| Degree | | 0-5 |
| Radical electrons | | Number of radical electrons of the atom |
| Chiral center | | Whether the atom is a chiral center |
| Chirality type | | ‘R’, ‘S’ |
| In aromatic ring | | Whether the atom is aromatic |

**Table S4.** The definition of initial AttentiveFP bond feature.

| **Features** | **Descriptions** |
| --- | --- |
| Conjugated | Whether the bond is conjugated. |
| In ring | Whether the bond is part of a ring. |
| Bond type | Single, double, triple, or aromatic. |
| Stereo | ‘Stereonone’, ‘Stereoany’, ‘Stereoz’, ‘Stereoe’,’Stereocis’, ‘Stereotrans’. |

**Table S5.** The PU learning analysis results of non-drug samples.

| **Name** | **Total** | **RN** | **Unlabeled** | **Drug-similar (%)** |
| --- | --- | --- | --- | --- |
| FDA_ZINC | 5,358 | 2,449 | 30 | 54.3 |
| Worlddrug_ChEMBL | 4,936 | 2,184 | 284 | 55.8 |
| Worlddrug_GDB17 | 4,936 | 2,452 | 16 | 50.3 |

**Table S6.** Data and model performance of the ADMET endpoints.

| **Endpoint (abb.)** | **Number** | **Accuracy** |
| --- | --- | --- |
| Ames mutagenesis (Ames) | 8,451 | 0.851 |
| Breast Cancer Resistance Protein inhibitor (BCRPi) | 970 | 0.845 |
| Bile Salt Export Pump inhibitor (BSEPi) | 607 | 0.827 |
| Caco-2 permeability (caco2) | 664 | 0.756 |
| Carcinogenicity (Carc) | 916 | 0.632 |
| Clearance (CL) | 636 | 0.629 |
| Genotoxicity (Gene) | 576 | 0.871 |
| Human either-a-go-go (hERG) | 3,022 | 0.914 |
| Hepatotoxicity (Hepa) | 2,544 | 0.722 |
| Human intestine absorption (HIA) | 578 | 0.934 |
| Human oral bioavailability (HOB) | 995 | 0.706 |
| Drug induced nephrotoxicity (DIN) | 777 | 0.738 |
| Mitochondrial toxicity (MMP) | 3,407 | 0.841 |
| OATP1B1 inhibitor (OATP1B1i) | 1,855 | 0.894 |
| OATP1B3 inhibitor (OATP1B3i) | 1,873 | 0.928 |
| OCT2 inhibitor (OCT2i) | 904 | 0.805 |
| P-glycoprotein inhibitor (P-gpi) | 1,944 | 0.864 |
| P-glycoprotein substrate (P-gps) | 1,564 | 0.811 |
| Reproductive toxicity (Repro) | 1,823 | 0.845 |
| Rat oral acute toxicity (ROA) | 4,068 | 0.818 |

Details of each endpoint are available in admetSAR2.0 (http://lmmd.ecust.edu.cn/admetsar2/about/models).

**Table S7.** The SHAP value analysis for the ADMET endpoints.

| **Endpoint** | **SHAP Value** | **Importance** |
| --- | --- | --- |
| Hepa | 1.000 | 0.045 |
| BCRPi | 0.088 | 0.022 |
| Carc | 0.096 | 0.022 |
| Gene | 0.089 | 0.013 |
| hERG | 0.175 | 0.011 |
| P_pgi | 0.065 | 0.008 |
| OCT2i | 0.092 | 0.008 |
| BSEPi | 0.089 | 0.006 |
| CL | 0.248 | 0.003 |
| HIA | 0.088 | 0.000 |
| Caco2 | 0.183 | -0.003 |
| ROA | 0.354 | -0.009 |
| Ames | 0.450 | -0.010 |
| MMP | 1.339 | -0.023 |
| OATP1B3i | 1.238 | -0.027 |
| P_pgs | 0.186 | -0.048 |
| OATP1B1i | 0.434 | -0.054 |
| DIN | 0.241 | -0.059 |
| HOB | 0.467 | -0.092 |
| Repro | 0.412 | -0.099 |

**Table S8.** Traditional machine learning model parameters.

| **Methods** | **Representation** | **Parameters** |
| --- | --- | --- |
| SVM | Fingerprints | {'C': 8.0, 'class_weight': 'balanced', 'gamma': 0.03125, 'kernel': 'rbf'} |
|  | Descriptors | {'C': 2.0, 'class_weight': 'balanced', 'gamma': 0.0001, 'kernel': 'rbf'} |
|  | ADMET profile | {'C': 2.0, 'class_weight': 'balanced', 'gamma': 0.5, 'kernel': 'rbf'} |
|  | Property profile | {'C': 128, 'class_weight': 'balanced', 'gamma': 0.125, 'kernel': 'rbf'} |
| GBM | Fingerprints | {'max_depth': 7, 'min_child_samples': 8, 'num_leaves': 19} |
|  | Descriptors | {'max_depth': 7, 'min_child_samples': 14, 'num_leaves': 17} |
|  | ADMET profile | {'max_depth': 7, 'min_child_samples': 4, 'num_leaves': 19} |
|  | Property profile | {'max_depth': 7, 'min_child_samples': 14, 'num_leaves': 13} |
| LR | QED | {'C': 0.03125, 'penalty': 'l2'} |
|  |  |  |

**Table S9.** Graph neural network model parameters.

| **Methods** | **Parameters** |
| --- | --- |
| GCN | "batch_size":256,"lr":0.0003,"predictor_hidden_feats":256,"residual": false, "weight_decay": 0.002 |
| GAT | alpha: 0.240, "batch_size": 64, "gnn_hidden_feats": 128, "lr": 0.0006,"num_heads":8,"predictor_hidden_feats":128,"weight_decay": 0.0024 |
| GraphSAGE | "aggregator_type":"lstm","batch_size":256,"gnn_hidden_feats": 64, "lr": 0.0879, "weight_decay": 0.0002 |
| AttentiveFP | "batch_size":128,"graph_feat_size":64,"lr":0.003,"num_timesteps": 2, "weight_decay": 4.358e-05 |

**Table S10.** Impact of feature normalization on the model.

| **Representation** | **Accuracy** | **Precision** | **Recall** | **AUC** | **SP** |
| --- | --- | --- | --- | --- | --- |
| Descriptor_Norm | 0.970±0.003 | 0.968±0.004 | 0.973±0.002 | 0.995±0.001 | 0.968±0.004 |
| Descriptor | 0.752±0.003 | 0.939±0.006 | 0.538±0.002 | 0.785±0.008 | 0.965±0.003 |

**Table S11.** The ten-fold cross-validation results for all models.

| **Model** | **Accuracy** | **Precision** | **Recall** | **AUC** | **SP** |
| --- | --- | --- | --- | --- | --- |
| GBM_Descriptor | 0.968±0.001 | 0.965±0.003 | 0.972±0.001 | 0.994±0.000 | 0.964±0.003 |
| SVM_Descriptor | 0.970±0.003 | 0.968±0.004 | 0.973±0.002 | 0.995±0.001 | 0.968±0.004 |
| SVM_TopoTorsion | 0.949±0.000 | 0.945±0.001 | 0.953±0.001 | 0.990±0.000 | 0.945±0.001 |
| SVM_RDKFingerprint | 0.955±0.002 | 0.952±0.002 | 0.958±0.001 | 0.992±0.000 | 0.952±0.002 |
| SVM_Morgan | 0.975±0.001 | 0.975±0.002 | 0.976±0.001 | 0.996±0.000 | 0.975±0.002 |
| SVM_MACCS | 0.974±0.003 | 0.974±0.004 | 0.975±0.002 | 0.995±0.001 | 0.974±0.004 |
| SVM_AtomPairs | 0.971±0.001 | 0.969±0.003 | 0.973±0.001 | 0.994±0.001 | 0.968±0.003 |
| GBM_RDKFingerprint | 0.922±0.003 | 0.910±0.003 | 0.936±0.004 | 0.975±0.001 | 0.908±0.003 |
| GBM_Morgan | 0.950±0.002 | 0.945±0.002 | 0.955±0.002 | 0.989±0.001 | 0.944±0.002 |
| GBM_MACCS | 0.972±0.002 | 0.976±0.002 | 0.967±0.002 | 0.994±0.000 | 0.976±0.002 |
| GBM_AtomPairs | 0.972±0.002 | 0.971±0.002 | 0.972±0.001 | 0.995±0.000 | 0.971±0.002 |
| GBM_TopoTorsion | 0.962±0.001 | 0.957±0.001 | 0.969±0.001 | 0.991±0.001 | 0.956±0.001 |
| GraphSAGE | 0.869±0.170 | 0.981±0.016 | 0.757±0.356 | 0.988±0.010 | 0.981±0.016 |
| GCN | 0.901±0.045 | 0.849±0.074 | 0.984±0.016 | 0.988±0.002 | 0.818±0.104 |
| GAT | 0.954±0.021 | 0.959±0.026 | 0.949±0.021 | 0.990±0.006 | 0.959±0.026 |
| AttentiveFP | 0.833±0.035 | 0.790±0.060 | 0.913±0.019 | 0.895±0.052 | 0.752±0.089 |
| LR_QED | 0.627±0.004 | 0.637±0.005 | 0.591±0.001 | 0.684±0.002 | 0.663±0.007 |
| GBM_ADMET Profile_Prob | 0.984±0.000 | 0.992±0.000 | 0.975±0.000 | 0.996±0.000 | 0.992±0.000 |
| GBM_ADMET Profile_Pred | 0.834±0.003 | 0.846±0.010 | 0.815±0.007 | 0.903±0.003 | 0.852±0.013 |
| GBM_ADMET Profile_Prob | 0.984±0.002 | 0.989±0.003 | 0.979±0.001 | 0.997±0.000 | 0.989±0.003 |
| GBM_ADMET Profile_Pred | 0.824±0.002 | 0.844±0.002 | 0.795±0.004 | 0.895±0.001 | 0.853±0.002 |
| SVM_ADMET Profile_Prob | 0.947±0.001 | 0.958±0.001 | 0.936±0.001 | 0.983±0.001 | 0.959±0.001 |
| SVM_ADMET Profile_Pred | 0.778±0.003 | 0.764±0.026 | 0.807±0.044 | 0.812±0.012 | 0.749±0.048 |
| SVM_ADMET Profile_Prob | 0.959±0.002 | 0.965±0.002 | 0.953±0.003 | 0.987±0.001 | 0.966±0.002 |
| SVM_ADMET Profile_Pred | 0.693±0.003 | 0.748±0.012 | 0.582±0.023 | 0.714±0.022 | 0.804±0.021 |
| SVM_Property Profile_Prob | 0.977±0.001 | 0.986±0.001 | 0.968±0.001 | 0.997±0.000 | 0.986±0.001 |
| SVM_Property Profile_Pred | 0.888±0.002 | 0.890±0.002 | 0.886±0.006 | 0.950±0.001 | 0.890±0.002 |
| SVM_Property Profile_Prob | 0.976±0.002 | 0.980±0.002 | 0.972±0.005 | 0.996±0.000 | 0.980±0.002 |
| SVM_Property Profile_Pred | 0.887±0.001 | 0.896±0.001 | 0.875±0.003 | 0.949±0.003 | 0.899±0.001 |
| GBM_Property Profile_Prob | 0.987±0.001 | 0.991±0.001 | 0.983±0.002 | 0.998±0.000 | 0.991±0.001 |
| GBM_Property Profile_Pred | 0.903±0.001 | 0.905±0.002 | 0.899±0.003 | 0.961±0.001 | 0.906±0.002 |
| GBM_Property Profile_Prob | 0.987±0.001 | 0.991±0.002 | 0.982±0.001 | 0.998±0.000 | 0.991±0.002 |
| GBM_Property Profile_Pred | 0.898±0.002 | 0.908±0.002 | 0.884±0.002 | 0.959±0.001 | 0.911±0.002 |

**Table S12.** The test set results for all models.

| **Model** | **Accuracy** | **Precision** | **Recall** | **AUC** | **SP** |
| --- | --- | --- | --- | --- | --- |
| GBM_Descriptor | 0.969±0.003 | 0.968±0.004 | 0.969±0.001 | 0.995±0.000 | 0.968±0.004 |
| SVM_Descriptor | 0.968±0.002 | 0.970±0.007 | 0.967±0.003 | 0.995±0.001 | 0.970±0.007 |
| GBM_RDKFingerprint | 0.919±0.006 | 0.917±0.009 | 0.922±0.003 | 0.977±0.003 | 0.917±0.009 |
| GBM_Morgan | 0.946±0.006 | 0.952±0.010 | 0.938±0.002 | 0.989±0.003 | 0.953±0.01 |
| GBM_MACCS | 0.977±0.004 | 0.980±0.004 | 0.974±0.004 | 0.997±0.001 | 0.980±0.004 |
| GBM_AtomPairs | 0.972±0.002 | 0.973±0.005 | 0.971±0.002 | 0.996±0.001 | 0.973±0.005 |
| GBM_TopoTorsion | 0.963±0.004 | 0.961±0.009 | 0.965±0.002 | 0.993±0.001 | 0.961±0.010 |
| SVM_RDKFingerprint | 0.953±0.005 | 0.954±0.007 | 0.952±0.007 | 0.991±0.002 | 0.954±0.008 |
| SVM_Morgan | 0.957±0.006 | 0.957±0.008 | 0.957±0.004 | 0.994±0.002 | 0.957±0.008 |
| SVM_MACCS | 0.981±0.001 | 0.978±0.003 | 0.984±0.001 | 0.997±0.001 | 0.978±0.003 |
| SVM_AtomPairs | 0.975±0.002 | 0.977±0.005 | 0.973±0.002 | 0.997±0.001 | 0.977±0.005 |
| SVM_TopoTorsion | 0.974±0.003 | 0.970±0.007 | 0.978±0.002 | 0.995±0.001 | 0.970±0.007 |
| GraphSAGE | 0.869±0.150 | 0.963±0.032 | 0.776±0.332 | 0.989±0.002 | 0.962±0.033 |
| GCN | 0.905±0.034 | 0.848±0.053 | 0.992±0.005 | 0.991±0.002 | 0.818±0.074 |
| GAT | 0.961±0.008 | 0.951±0.006 | 0.972±0.011 | 0.992±0.003 | 0.949±0.006 |
| AttentiveFP | 0.861±0.033 | 0.834±0.070 | 0.915±0.087 | 0.928±0.049 | 0.807±0.111 |
| LR_QED | 0.637±0.001 | 0.648±0.001 | 0.598±0.001 | 0.681±0.002 | 0.676±0.001 |
| GBM_ADMET Profile_Prob | 0.853±0.006 | 0.989±0.005 | 0.714±0.008 | 0.958±0.003 | 0.992±0.004 |
| GBM_ADMET Profile_Pred | 0.831±0.009 | 0.847±0.008 | 0.807±0.012 | 0.896±0.007 | 0.854±0.008 |
| GBM_ADMET Profile_Prob | 0.798±0.001 | 0.982±0.005 | 0.606±0.001 | 0.943±0.002 | 0.989±0.003 |
| GBM_ADMET Profile_Pred | 0.809±0.008 | 0.843±0.009 | 0.760±0.010 | 0.884±0.005 | 0.859±0.008 |
| SVM_ADMET Profile_Prob | 0.829±0.002 | 0.948±0.004 | 0.696±0.003 | 0.905±0.005 | 0.962±0.003 |
| SVM_ADMET Profile_Pred | 0.780±0.015 | 0.766±0.014 | 0.810±0.069 | 0.819±0.015 | 0.751±0.039 |
| SVM_ADMET Profile_Prob | 0.793±0.008 | 0.947±0.005 | 0.620±0.020 | 0.896±0.010 | 0.965±0.004 |
| SVM_ADMET Profile_Pred | 0.695±0.002 | 0.760±0.007 | 0.570±0.007 | 0.735±0.002 | 0.820±0.009 |
| SVM_Property Profile_Prob | 0.892±0.004 | 0.980±0.003 | 0.801±0.009 | 0.966±0.001 | 0.984±0.002 |
| SVM_Property Profile_Pred | 0.876±0.014 | 0.893±0.018 | 0.854±0.011 | 0.944±0.008 | 0.898±0.018 |
| SVM_Property Profile_Prob | 0.856±0.007 | 0.971±0.007 | 0.734±0.021 | 0.946±0.004 | 0.978±0.006 |
| SVM_Property Profile_Pred | 0.871±0.005 | 0.899±0.011 | 0.836±0.007 | 0.937±0.005 | 0.906±0.012 |
| GBM_Property Profile_Prob | 0.867±0.004 | 0.990±0.003 | 0.741±0.005 | 0.975±0.004 | 0.992±0.002 |
| GBM_Property Profile_Pred | 0.893±0.003 | 0.910±0.007 | 0.873±0.004 | 0.957±0.004 | 0.914±0.008 |
| GBM_Property Profile_Prob | 0.812±0.006 | 0.984±0.004 | 0.635±0.013 | 0.952±0.002 | 0.989±0.002 |
| GBM_Property Profile_Pred | 0.883±0.009 | 0.917±0.012 | 0.842±0.006 | 0.948±0.003 | 0.924±0.011 |

**Table S13.** P values of DBPP predictor on various data sets.

| **Data set** | **P value** |
| --- | --- |
| Drugs & ZINC | $\to0$ |
| Drugs & ChEMBL | $\to0$ |
| Drugs & GDB17 | $\to0$ |
| Drugs & Investigation | 1.31E-131 |
| Drugs & Withdrawn | 4.83E-23 |
| ZINC & Investigation | $\to0$ |
| ZINC & Withdrawn | 9.00E-163 |
| ZINC & ChEMBL | $\to0$ |
| ZINC & GDB17 | $\to0$ |
| Withdrawn & Investigation | 2.00E-75 |
| Withdrawn & ChEMBL | 1.51E-118 |
| Withdrawn & GDB17 | 3.34E-151 |
| Investigation & ChEMBL | 1.20E-47 |
| Investigation & GDB17 | 1.09E-247 |
| ChEMBL & GDB17 | $\to0$ |

**Table S14.** Data set information of the data set for score analysis.

| **Name** | **Type** | **Numbers** |
| --- | --- | --- |
| Data set | Drug | 200 |
|  | ZINC | 200 |
|  | ChEMBL | 100 |
|  | GDB17 | 100 |
|  | TCMSP | 100 |
|  | Investigation | 100 |

| **Compound ID** | **DBPP Score** | **Compound ID** | **DBPP Score** |
| --- | --- | --- | --- |
| 11a | 0.2998 | 19b | 0.7386 |
| 11b | 0.0118 | 21a | 0.8131 |
| 11c | 0.2074 | 20 | 0.7140 |
| 11d | 0.3880 | 23 | 0.5305 |
| 11e | 0.4047 | 27 | 0.6353 |
| 12 | 0.5457 | 26 | 0.4909 |
| 11f | 0.0810 | 30 | 0.5750 |
| 11g | 0.2212 | 21b | 0.8391 |
| 14a | 0.5137 | 21c | 0.5770 |
| 11h | 0.2975 | 21d | 0.6775 |
| 11i | 0.3668 | 21e | 0.8767 |
| 11j | 0.4230 | 21f | 0.5904 |
| 11k | 0.5034 | 34a | 0.8598 |
| 11l | 0.3532 | 34b | 0.8824 |
| 11m | 0.7637 | 37 | 0.4947 |
| 11n | 0.6824 | 36 | 0.5633 |
| 11o | 0.3959 | 52a | 0.7768 |
| 11p | 0.4297 | 52b | 0.8877 |
| 11q | 0.6600 | 52c | 0.7944 |
| 14b | 0.3148 | 52d | 0.9187 |
| 14c | 0.3461 | 52e | 0.8665 |
| 14d | 0.6676 | 52f | 0.8887 |
| 11r | 0.2936 | 52g | 0.7242 |
| 16 | 0.2097 | 52h | 0.9086 |
| R-11d | 0.3880 | 52i | 0.8954 |
| 19a | 0.3880 | R-52b | 0.8877 |

**Table S15.** DBPP scores of the 52 molecules in case study.

**Text S1.** The equation of correlation analysis.

|  | $r =\left\vert\frac{\sum_{i=1}^{N} \left( x_{i}-\bar{x} \right)\left( y_{i}-\bar{y} \right)}{\sqrt{\sum_{i=1}^{N} \left( x_{i}-\bar{x} \right)^{2}}\sqrt{\sum_{i=1}^{N} \left( y_{i}-\bar{y} \right)^{2}}} \right\vert$ | **(1)** |
| --- | --- | --- |

where $N$ is the number of samples, $x$ and $y$ represent the value of the endpoints, while $\bar{x}$ and $\bar{y}$ are the mean of the features.


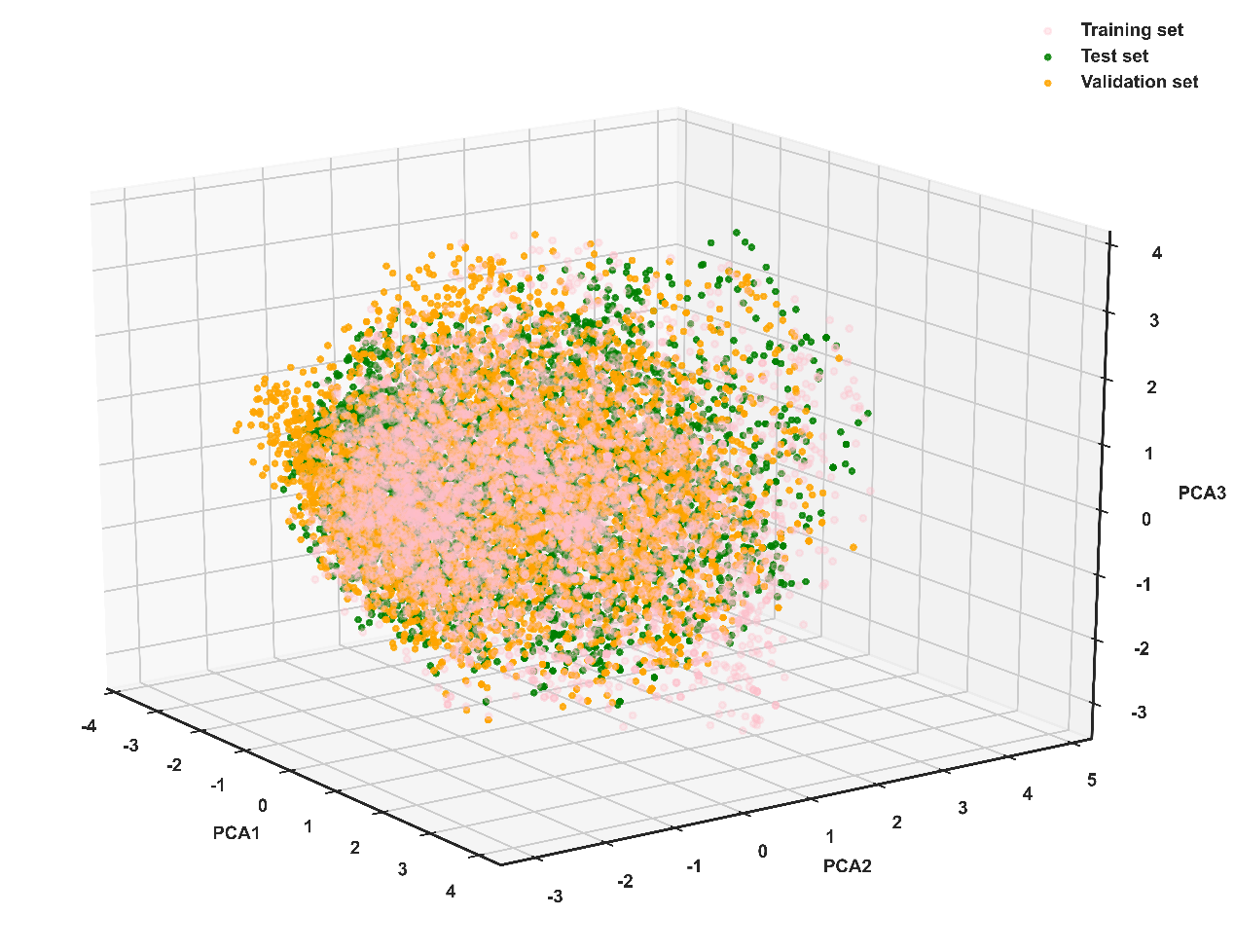


**Figure S1.** Three-Dimensional principal component analysis on the training, test and validation set.

**
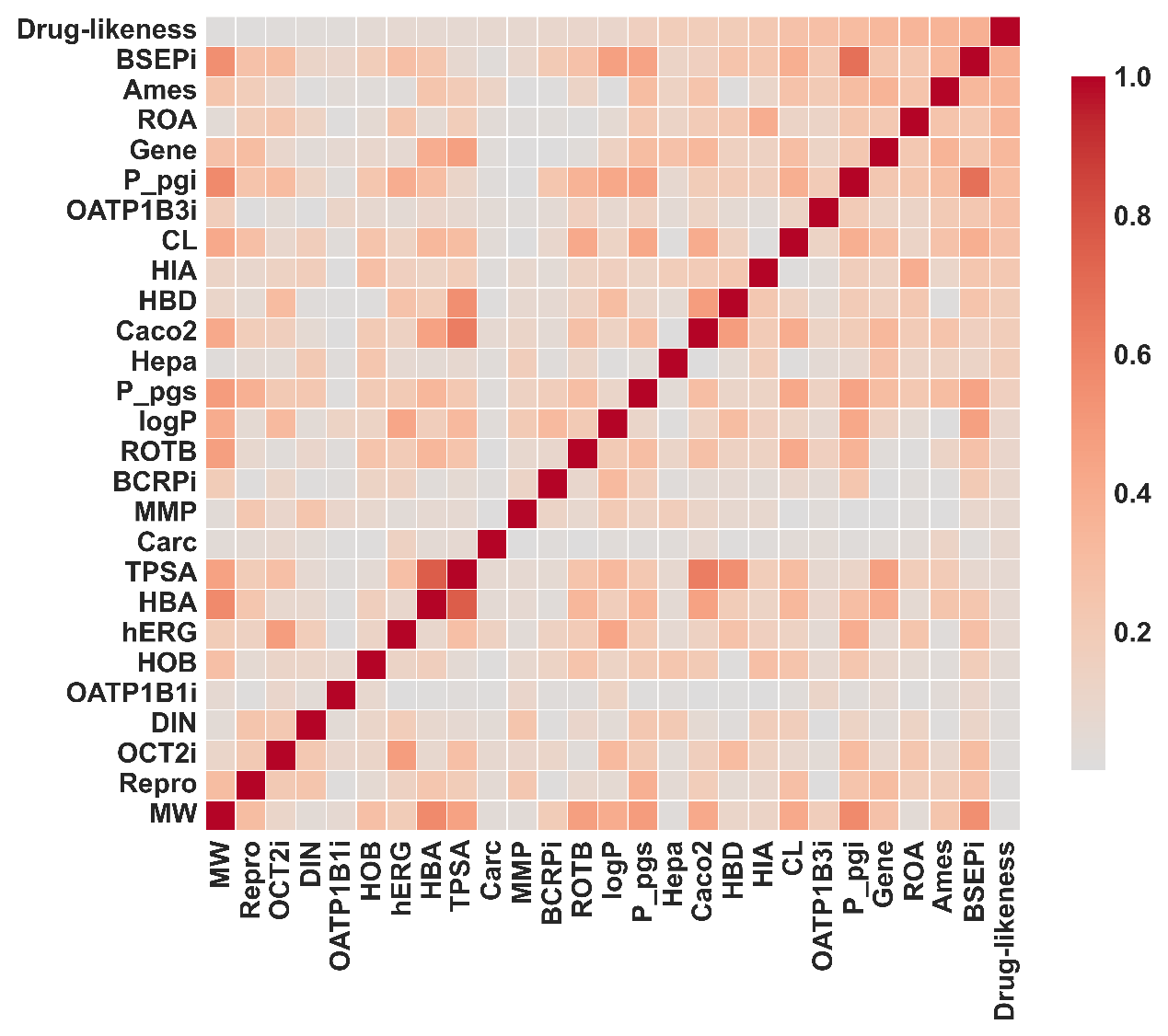
**

**Figure S2.** Heat map of property profiles endpoints and drug-likeness correlation analysis.


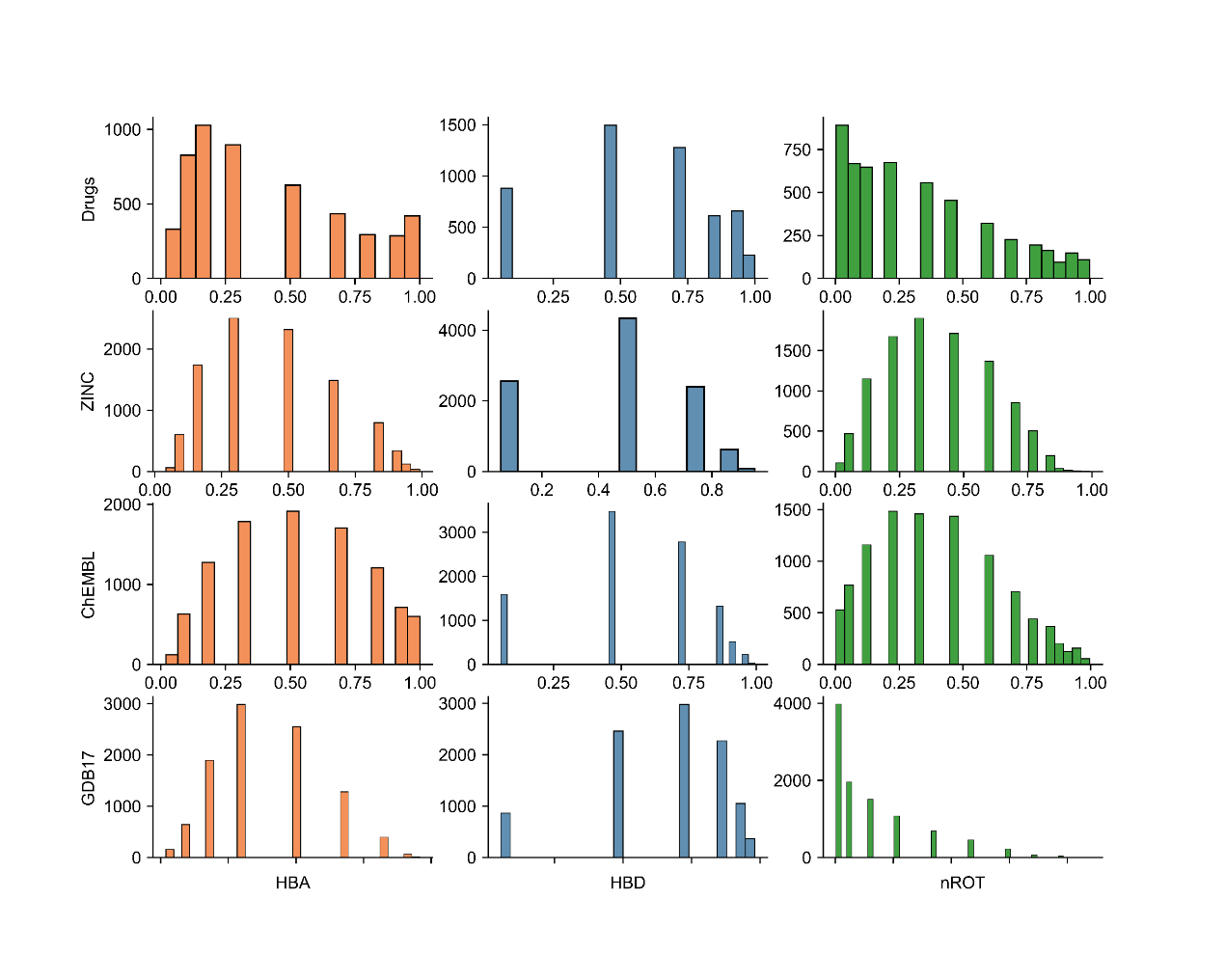


**Figure S3.** Physicochemical property profiles of drugs and nondrugs samples (ZINC, ChEMBL and GDB17) correlation analysis, including HBA, HBD and nROT.


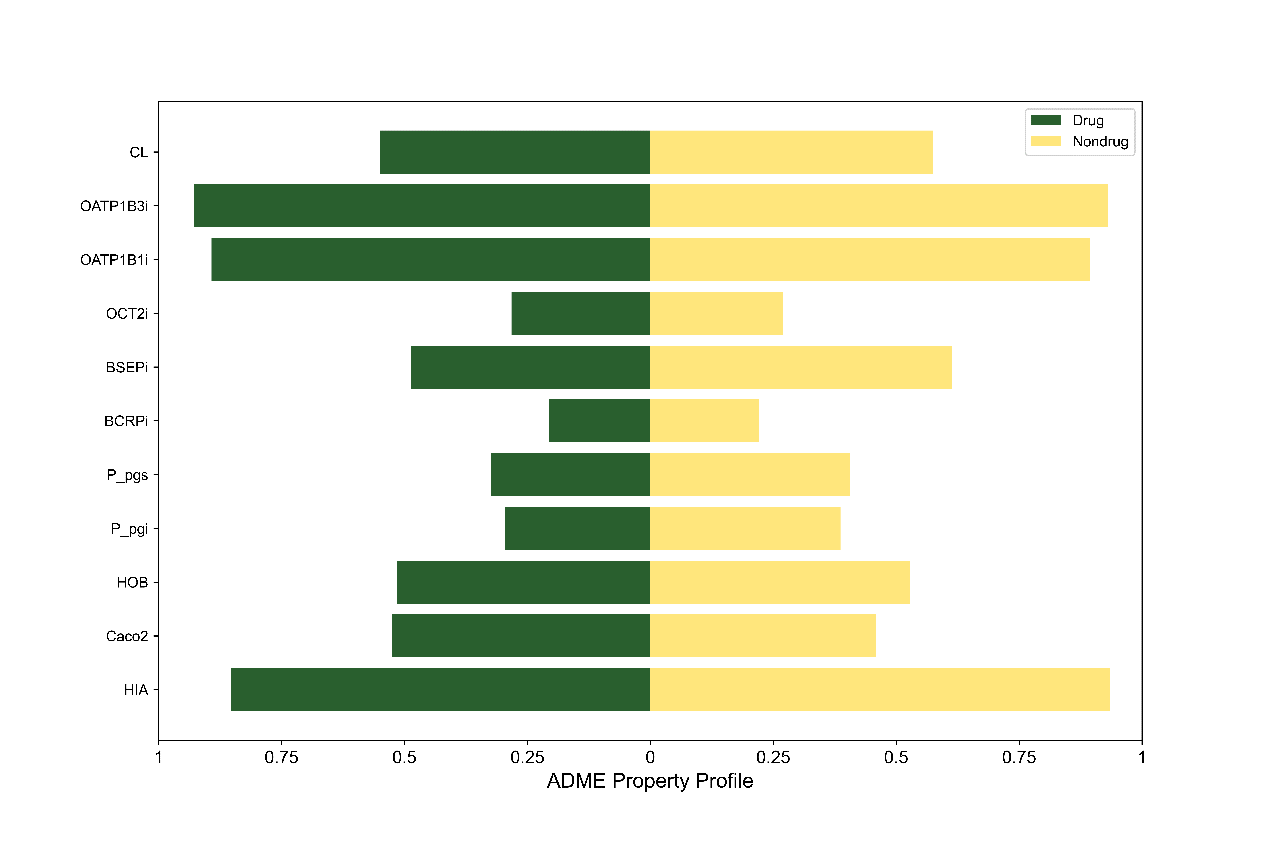


**Figure S4.** Plot of ADME property profiles analysis for drugs and nondrugs molecules.


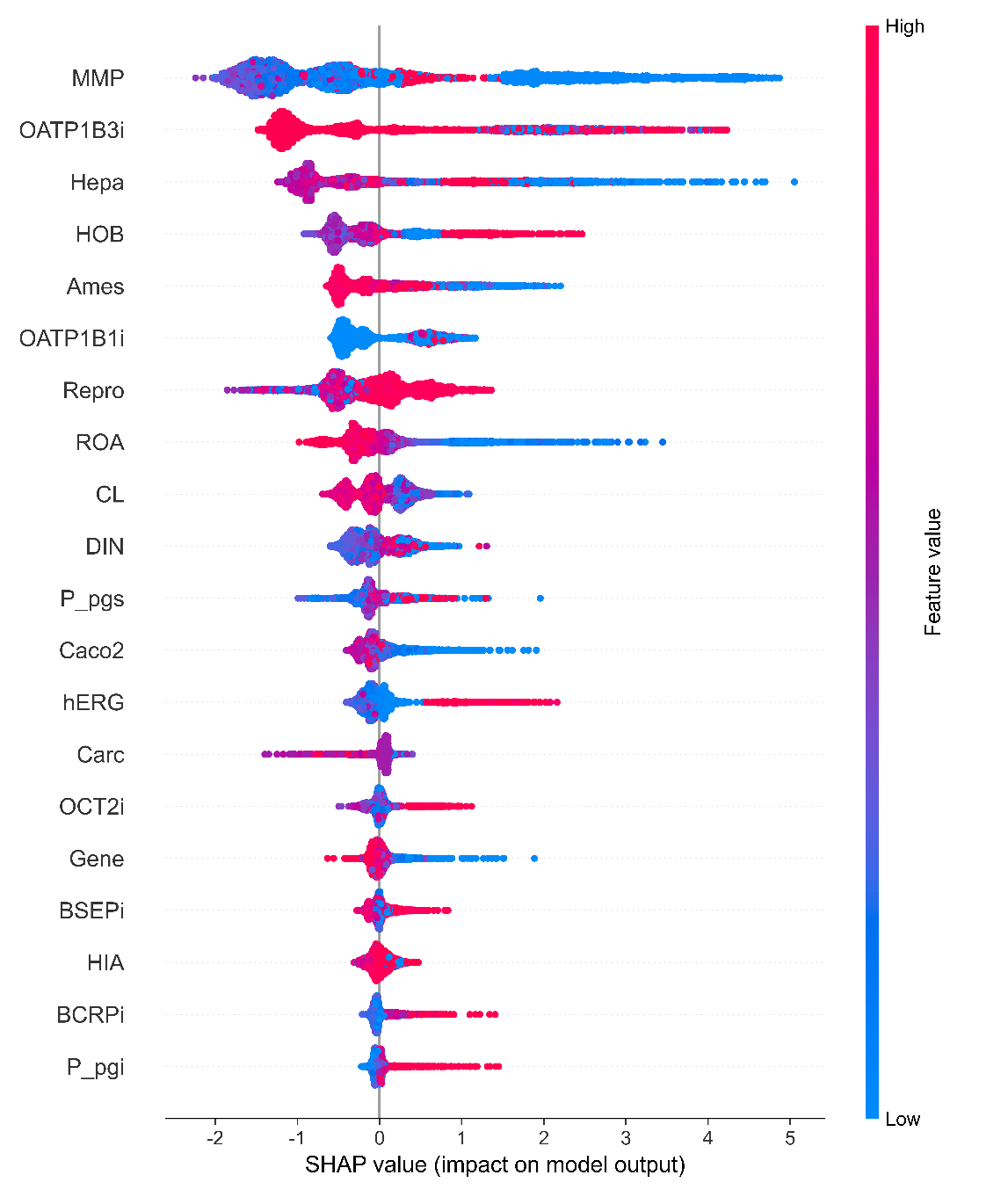


**Figure S5.** Analysis of the SHAP values for ADMET endpoints.


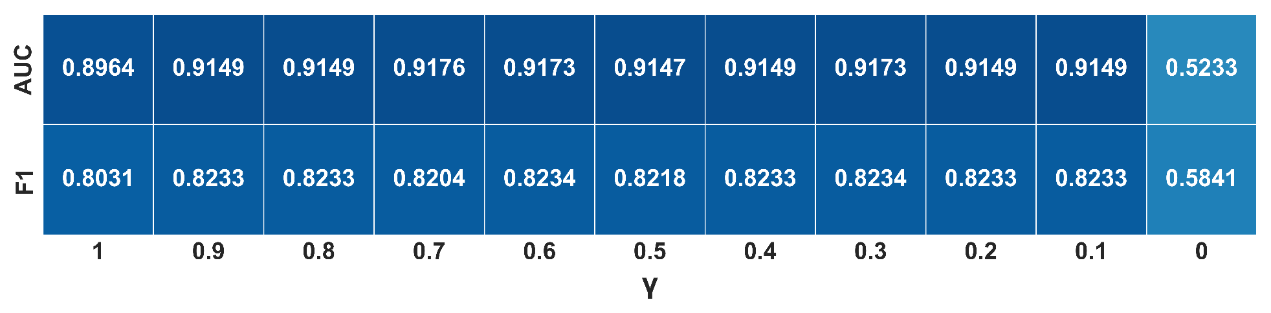


**Figure S6.** The performance of DBPP model corresponding to different values of γ.
